# Outcomes and Safety of Revascularization Approaches for Stroke Related to Isolated Vertebral Artery Occlusions (BRAVO)

**DOI:** 10.1161/STROKEAHA.125.051675

**Published:** 2026-03-10

**Authors:** Alexander Salerno, Vincent Dunet, Marek Sykora, Philipp Baumgartner, Visnja Padjen, Guido Bigliardi, Dylan Ryan, Mirjam R. Heldner, Christian H. Nolte, Sami Curtze, Andrea Zini, Michael Nahhas, Thanh N. Nguyen, Henrik Gensicke, Lars Kellert, Jong S. Kim, Christian Boehme, James E. Siegler, Paolo Machi, Joao Pedro Marto, Alessandro Pezzini, Jens Fiehler, Margareta Abrahamson, Stefan Krebs, Marialuisa Zedde, Jiangyong Min, Ronen Leker, Carlo W. Cereda, Carlos A. Molina, Charlotte Cordonnier, Volker Puetz, Bianca Mazini, Guillaume Saliou, Stefan T. Engelter, Davide Strambo, Patrik Michel

**Affiliations:** Stroke Center, Neurology Service, Department of Clinical Neurosciences, Lausanne University Hospital and University of Lausanne, Switzerland (A.S., D.S., P. Michel).; Department of Diagnostic and Interventional Radiology, Lausanne University Hospital and University of Lausanne, Switzerland (V.D., B.M., G.S.).; Department of Neurology, St. Johns Hospital, Vienna, Austria (M.S., S.K.).; Department of Neurology, University Hospital of Zürich and University of Zurich, Switzerland (P.B.).; Neurology Clinic, University Clinical Centre of Serbia, Belgrade (V. Padjen).; Stroke Unit, Neurology Clinic, Ospedale Civile di Baggiovara, AOU Modena, Italy (G.B.).; Department of Neurology, Duke University School of Medicine, Durham, NC (D.R.).; Stroke Unit, Department of Neurology, Inselspital, Bern University Hospital and University of Bern, Switzerland (M.R.H.).; Department of Neurology, Charité-Universitatmedizin Berlin, Germany (C.H.N.).; Department of Neurology, Helsinki University Hospital and Clinical Neurosciences, University of Helsinki, Finland (S.C.).; Department of Neurology and Stroke Center, IRCCS Istituto Delle Scienze Neurologiche di Bologna, Italy (A.Z.).; Department of Neurology, University of Texas McGovern Medical School, Houston (M.N.).; Neurology Department, Boston University Chobanian and Avedisian School of Medicine, MA (T.N.N.).; Neurology and Neurorehabilitation, University Department of Geriatric Medicine, Felix Platter and University Hospital Basel, Switzerland (H.G., S.T.E.).; Department of Neurology, Klinikum der Universität München, Ludwig-Maximilians-University, Munich, Germany (L.K.).; Department of Neurology, Gangneung Asan Hospital, University of Ulsan College of Medicine, Seoul, South Korea (J.S.K.).; Department of Neurology, Medical University of Innsbruck, Austria (C.B.).; Department of Neurology, Cooper Neurological Institute, Cooper University Hospital, Camden, NJ (J.E.S.).; Department of Neuroradiology, Geneva University Hospital, Switzerland (P. Machi).; Department of Neurology, Hospital de Egas Moniz, Centro Hospitalar Lisboa Ocidental, Lisbon, Portugal (J.P.M.).; Department of Medicine and Surgery, University of Parma and Stroke Care Program, Department of Emergency, Parma University Hospital, Italy (A.P.).; Department of Diagnostic and Interventional Neuroradiology, University Medical Center Hamburg-Eppendorf, Germany (J.F.).; Department of Neurology, Sahlgrenska University Hospital, Göteborg, Sweden (M.A.).; Neurology Unit, Stroke Unit, Azienda Unità Sanitaria Locale-IRCCS di Reggio Emilia, Italy (M.Z.).; Department of Neurosciences, Corewell Health West, Michigan State University, Grand Rapids (J.M.).; Department of Neurology, Hadassah-Hebrew University Medical Center, Jerusalem, Israel (R.L.).; Stroke Center EOC, Neurocenter of Southern Switzerland, Ospedale Regionale di Lugano, Ente Ospedaliero Cantonale, Lugano (C.W.C.).; Stroke Unit, Department of Neurology, Hospital Universitari Vall d'Hebron, Barcelona, Spain (C.A.M.).; Department of Neurology, University of Lille, Inserm, CHU Lille Lille Neuroscience & Cognition, France (C.C.).; Department of Neurology, University Clinics Carl Gustav Carus, Dresden, Germany (V. Puetz).

**Keywords:** cerebrovascular disorders, conservative treatment, ischemic stroke, thrombolytic therapy, cerebral arterial diseases, vertebral artery, thrombectomy

## Abstract

**BACKGROUND:**

The best revascularization strategy for acute ischemic stroke from isolated vertebral artery occlusion remains unclear.

**METHODS:**

This retrospective, international, multicenter cohort study included patients from 30 comprehensive stroke centers across Europe (n=23), North America (n=5), and Asia (n=2) between 2016 and 2022. Eligible patients presented with acute ischemic stroke within 24 hours of last seen well and had imaging-confirmed isolated vertebral artery occlusion. Two treatment comparisons were analyzed: intravenous thrombolysis (IVT)-only versus conservative treatment (Cx), and endovascular treatment (EVT)±IVT versus medical management (Cx and IVT). The primary outcome was the shift in 3-month modified Rankin Scale (mRS) score; secondary outcomes included early neurological improvement (24-hour-delta National Institutes of Health Stroke Scale score), recanalization, early neurological deterioration of ischemic origin, symptomatic intracerebral hemorrhage, and 3-month mortality. Analyses were adjusted using inverse probability of treatment weighting (IPTW).

**RESULTS:**

Among 494 patients, 143 (29%) received Cx, 218 (44%) IVT-only, and 133 (27%) EVT±IVT. Compared with Cx, IVT-only showed similar 3-month mRS score (IPTW-adjusted odds ratio [aOR] mRS shift score, 1.32 [95% CI, 0.80–2.18]), greater early neurological improvement (IPTW-adjusted-β coefficient, −1 [95% CI, −2.05 to 0.05]), and higher recanalization rates (IPTW-aOR, 4.33 [95% CI, 1.36–13.78]). Compared with MM (=IVT+Cx), EVT±IVT was associated with an unfavorable mRS shift score (IPTW-aOR mRS shift score, 0.51 [95% CI, 0.35–0.74]), higher early neurological deterioration of ischemic origin (IPTW-aOR, 9.06 [95% CI, 2.86–28.67]), and symptomatic intracerebral hemorrhage (IPTW-aOR, 6.05 [95% CI, 1.14–32.1]) though recanalization was over 4-fold higher (OR, 4.64 [95% CI, 1.90–11.33]). Patients with National Institutes of Health Stroke Scale score ≥10 showed point estimates favoring EVT+IVT (*P*_interaction_=0.025).

**CONCLUSIONS:**

IVT-only appeared safe and was associated with better early recovery and recanalization. EVT±IVT showed overall worse outcomes, potentially due to increased early neurological deterioration of ischemic origin and symptomatic intracerebral hemorrhage rates, but may confer benefit in moderate-to-severe strokes, warranting prospective trials in symptomatic isolated vertebral artery occlusion.

Despite robust evidence demonstrating the benefits of revascularization treatments for large vessel occlusions in anterior circulation strokes, the optimal acute management of occlusive posterior circulation (PC) acute ischemic strokes (AISs) other than basilar artery occlusion (BAO) remains less clear.

Approximately one-third of AISs in the PC are characterized by an arterial occlusion.^1^ Of these, isolated vertebral artery occlusions (iVAOs) represent the third most prevalent type, accounting for 18.5% of all PC occlusions. Their frequency ranks after multilevel occlusions (43.9%) and isolated posterior cerebral artery (PCA) occlusions (29.8%) but precede isolated BAOs (7.9%).^2^ Strokes associated with iVAO can present with minor deficits, as in a small posterior inferior cerebellar infarct, or severe clinical deficits caused by extensive infarction of the medulla, which may involve 1 or both corticospinal tracts.^3^ The extent of the initial deficits may, furthermore, be underestimated by commonly used neurological assessment scales, such as the National Institutes of Health Stroke Scale (NIHSS), a reason for a recent proposal for a specific posterior NIHSS (POST-NIHSS) score.^4^

Despite its significant burden, iVAO received the least attention among occlusive PC strokes.^5^ Although intravenous thrombolysis (IVT) has never been specifically tested in PC or iVAO strokes, indirect evidence suggests its safety and potential to improve 3-month functional outcome independently of the arterial occlusion pattern.^2,6–10^ For instance, the recent EXPECTS (Extending the Time Window for Thrombolysis in Posterior Circulation Stroke Without Early CT Signs) randomized controlled trial (RCT) demonstrated the efficacy and safety of IVT in minor PC strokes within the late window, including 30 of 234 (13%) patients with vertebral artery occlusions.

In contrast, the effect of endovascular treatments (EVT) in PC occlusions, particularly those other than BAO, remains poorly defined.^11^ While 2 recent RCTs established EVT’s benefit in moderate-to-severe BAO cases within 24 hours,^12–14^ thrombolysis rates in these studies were low.^12–14^ Conversely, trials such as DISTAL (Endovascular Therapy Plus Best Medical Treatment [BMT] Versus BMT Alone for Medium Vessel Occlusion Stroke) and ESCAPE-MeVO (Endovascular Treatment to Improve Outcomes for Medium Vessel Occlusions) found no added benefit of EVT over medical management (MM) for distal PCA occlusions.^15,16^

For iVAO, the only existing data on EVT derive from the analysis of a subgroup of 12 individuals with occlusion of the dominant vertebral artery enrolled in BEST (Endovascular Treatment Versus Standard Medical Treatment for Vertebrobasilar Artery Occlusion)^17^ and a recent descriptive registry analysis of 42 cases from a Chinese endovascular cohort.^18^ In the BEST trial, patients with iVAO seemed to have a worse outcome with EVT than MM defined as a combination of conservative treatment (Cx) and IVT treatment.

Our objective was to investigate the revascularization treatment outcome and safety of iVAO-related AIS in routine clinical practice. Two principal treatment comparisons were analyzed: (1) IVT versus Cx and (2) EVT±IVT versus MM (=Cx+IVT). The primary outcome was the 3-month modified Rankin Scale (mRS) shift score; secondary outcomes were ΔNIHSS score at 24 hours, V4 segment recanalization, early neurological deterioration of ischemic origin (ENDi), symptomatic intracerebral hemorrhage (sICH), and 3-month mortality.

## Methods

### Data Availability

The raw, anonymized data that support the findings of this study are available from the corresponding author upon reasonable request and after signing a data transfer and use agreement. If such data are used for a publication, its methods should be communicated, and internationally recognized authorship rules should be applied.

### Study Design

BRAVO (Best Revascularisation Approach for Posterior Circulation Strokes With Isolated Vertebral Artery Occlusions) was an investigator-initiated, retrospective, observational, global multicenter registry study of consecutive individuals aged ≥18 years who presented with an ischemic stroke attributed to an acute symptomatic and radiologically confirmed iVAO. Inclusion criteria, treatment groups, and primary and secondary outcomes were specified a priori and registered on ClinicalTrials.gov (Unique identifier: NCT05503212). For reporting, we followed the STROBE statement (Strengthening the Reporting of Observational Studies in Epidemiology) for observational studies,^19^ and the STROBE checklist is provided in Table S1. Data were contributed by 30 stroke centers (23 European, 5 North American, and 2 Asian). Patient inclusion spanned 2016 to 2022; center participation began within the EVA-TRISP (Endovascular Treatment and Thrombolysis for Ischemic Stroke Patients) network (Table S2) in 2022 and was subsequently expanded internationally.

### Population

Patients were included in the analysis if they presented with symptoms or signs of AIS within 24 hours of last seen well attributed by the treating physician to an acute vertebrobasilar ischemia secondary to an iVAO. The latter had to be confirmed by computed tomography angiography or magnetic resonance angiography. iVAO was defined as a complete absence of opacification (or flow void in the case of time-of-flight magnetic resonance angiography) either in any segment of the extracranial vertebral artery, of the intracranial vertebral artery, or both. Individuals with occlusion of both vertebral arteries, as assessed by each local investigator, were also included. We excluded individuals with a previously documented unilateral vertebral artery occlusion, an extension of the vertebral artery occlusion in any segment of the basilar artery, and those individuals with a simultaneous occlusion of the distal basilar and any PCA segment. However, if individuals had clinical or radiological signs of concomitant AIS in a territory other than the medulla and cerebellum, they were included if there was no other medium or large vessel PC occlusion visible on arterial imaging. Individuals whose AIS was considered nondisabling by the treating physician at the time of stroke, those with transient symptoms, and those with a missing 3-month mRS assessment were also excluded.

Data were retrospectively collected from stroke registries of high-volume EVT-capable stroke centers worldwide. Centers were contacted personally by the first and last authors. The inclusion period of the main analysis ranged from 2016 to 2022. Centers had to be able to provide information on consecutive individuals treated with EVT and IVT, and consecutive conservatively treated individuals (if available). A secondary analysis assessing the incidence of this kind of stroke was performed on a larger data set between 2003 and 2022. Participating centers retrospectively identified and submitted consecutive patients meeting the study inclusion and exclusion criteria, rather than screening all stroke admissions. Submitted cases were centrally verified for eligibility, and only those with complete key variables and available 3-month outcomes were retained for analysis. Figure S1 illustrates this submission and verification process, detailing final group allocation for the 2 main treatment contrasts.

A full list of the participating institutions and their respective enrollment period and contributions to treatment groups is given in Table S3. Part of the institutions was recruited from within the European EVA-TRISP Consortium.^20^

### Treatments

For the study, individuals were assigned to a respective treatment group, which could be Cx, IVT-only, or EVT±IVT. Individuals in the Cx group received standard of care treatment of the respective institution at the time of the stroke, such as single- or double-antiplatelet treatment (data not collected), but no IVT. For comparison with the EVT±IVT group, Cx treatment and IVT-only treatment were combined under the definition of MM. Revascularization strategies were decided by the treating physician according to local treatment practice at the time of stroke.

### Measures

The collected variables included information on demographics, cerebrovascular risk factors, clinical-radiological characteristics, time metrics, revascularization treatment, and safety-related outcomes. These are detailed in Table S4.

### Outcomes

The primary outcome of the study was defined as a significant shift toward better functional outcome on the ordinal mRS score at 3 months. Functional outcome data on mRS score were routinely collected either from in-person visits or by a structured phone call performed by trained personnel at each participating site, unblinded to the acute revascularization treatment performed. The secondary outcomes were early neurological improvement (delta NIHSS score at 24 hours), excellent 3-month outcome defined as mRS score of 0 to 1 or return to prestroke mRS score; good functional outcome, defined as mRS score of 0 to 2 or return to prestroke mRS score; sICH according to ECASS (European Cooperative Acute Stroke Study)-2 criteria, therefore not including subarachnoid hemorrhage; ENDi,^21^ defined as a worsening of at least 4 points on the NIHSS score within 24 hours without parenchymal hemorrhage on follow-up imaging or another identified cause; and 3-month mortality. Extracranial and intracranial vertebral artery recanalization was assessed on the first available computed tomography angiography or magnetic resonance angiography within 48 hours of last seen well or on ultrasound Doppler when these were not performed. Recanalization was classified as none, partial (50%–99% residual stenosis), or complete recanalization.

### Statistical Analysis

We compared baseline and outcome variables between individuals receiving IVT-only and those receiving Cx, and between individuals undergoing EVT±IVT and those receiving MM. We reported baseline descriptive statistics for the entire cohort and according to treatment groups. Continuous variables were expressed as median and interquartile range (IQR), and categorical variables as frequency and percentage.

To account for imbalance in potential confounders for the associations between treatment groups and outcomes, we used the inverse probability of treatment weighting (IPTW) approach as the main analysis. The estimation of propensity score weights was performed using the generalized boosted modeling method implemented in the TWANG package in R,^22^ a machine learning multivariable nonparametric regression technique that estimates the propensity score of individuals iteratively to maximize balance in observed covariates. The standardized mean difference (ES-mean) statistic was used as a stopping criterion for assessing and summarizing balance across pretreatment variables. A 10 000-tree generalized boosted modeling model with an interaction depth of 3, a shrinkage value of 0.01, and a bag fraction of 1 was utilized. To keep information on all patients, no trimming of outliers or nonoverlapping scores was performed, as stabilized IPTW downweights extreme values and minimizes their influence. The model included baseline clinical and radiological variables known to be associated with outcomes, such as biological sex, age, prestroke mRS score, admission blood glucose, admission NIHSS score, clinical-radiological other brainstem lesion, unilateral extracranial occlusion, multiple segments occlusion, TOAST classification (Trial of ORG 10172 in Acute Stroke Treatment), last seen well to hospital arrival delay, year of stroke onset, IVT, and EVT.

The balance of baseline characteristics between treatment groups was assessed before and after propensity score weighting by calculation of absolute standardized mean differences. An absolute standardized mean difference ≤10% was considered acceptable. The association between treatment group and each outcome was estimated through odds ratios and 95% CIs, calculated in univariable regression models with IPTW. Ordinal regression was used for the primary outcome, binary logistic regression models for the secondary binary outcome, and quantile regression for early neurological improvement. For all these outcomes, we also performed multivariable regression analysis as an alternative model correcting for the same confounders. We assessed potential heterogeneity in the effectiveness of EVT depending on preselected clinical-radiological features. These were analyzed by entering in the regression model for the primary outcome with IPTW, an interaction term between the treatment variable and the clinical-radiological feature of interest, and *P* values of the interaction term (*P*_interaction_) were calculated. Given the potential clustering effect of individuals from the same center, we included the referring center in each model as a cluster-level variable and calculated cluster-robust standard errors. To account for the evolution of revascularization treatment over time, the year of the stroke was also included in the statistical models. To account for missing data of the covariates, we performed multiple imputation by chained equations, generating 10 imputed data sets. We performed analyses on each imputed data set, and then, the estimates and the standard errors of the 10 imputed analyses were combined using the Rubin rules. The rate of missing data for each variable in the registry was reported in Table S5. All tests were 2-sided, and *P*<0.05 was considered significant. We performed statistical analysis with R statistical software, version 4.0.3.

### Ethics

Each center providing data was responsible for following their national and local ethical rules and regulations regarding the need for individual informed consent, data collection, storage, handling, and transmission, and for obtaining approval from their respective local authorities and ethics committees if necessary. Data were shared in anonymized or coded ways, respecting the current regulations in terms of data sharing and data protection.

## Results

### Population Characteristics

Participating institutions and the number of individuals recruited per center are provided in Table S3. To estimate the incidence of iVAO among all AISs, we performed an analysis on 13 centers that were able to provide further clinical data from their center on consecutive patients independently from revascularization treatment in the 2003 to 2022 time window. iVAO represented 351 of 43 385 (0.8%) of all AISs admitted within 24 hours of last seen well and 3.4% of all arterial occlusions. This corresponded to 5.5% of all PC 233 strokes and 17.7% of all PC occlusions.

From the initially received 819 individuals from 30 centers across Europe (23), North America (5), and Asia (2), we verified and excluded 79 patients because of a last seen well to arrival delay, which was superior to 24 hours or unavailable, 29 patients for missing 3-month mRS score, 135 for being in the time window between 2003 and 2015 because of significant improvement of EVT techniques after the introduction of stent retrievers, and finally 82 who did not undergo revascularization treatment because the stroke was not considered disabling according to the treating physician at the time of stroke management (see the exclusion flowchart in Figure S1).

The final data set for the analysis included 494 individuals, of whom 143 (29%) were treated with Cx, 218 (44%) with IVT-only, and 133 (27%) with EVT±IVT. Tables 1 and 2 show the baseline characteristics of the study. Figure S2A through S2D illustrates the distribution of covariates in both the disability and recanalization models for IVT and EVT cohorts, as well as propensity score distribution (Figure S3A through S3D). In all models, IPTW substantially improved balance, with most covariates falling below the conventional threshold for absolute standardized mean difference ≤10% after weighting, remaining within acceptable limits. A limited number of covariates, such as NIHSS score on admission, last seen well to arrival delays, and unilateral extracranial localization of occlusion, retained an absolute standardized mean difference >10%, indicating minor residual imbalance.

**Table 1.**
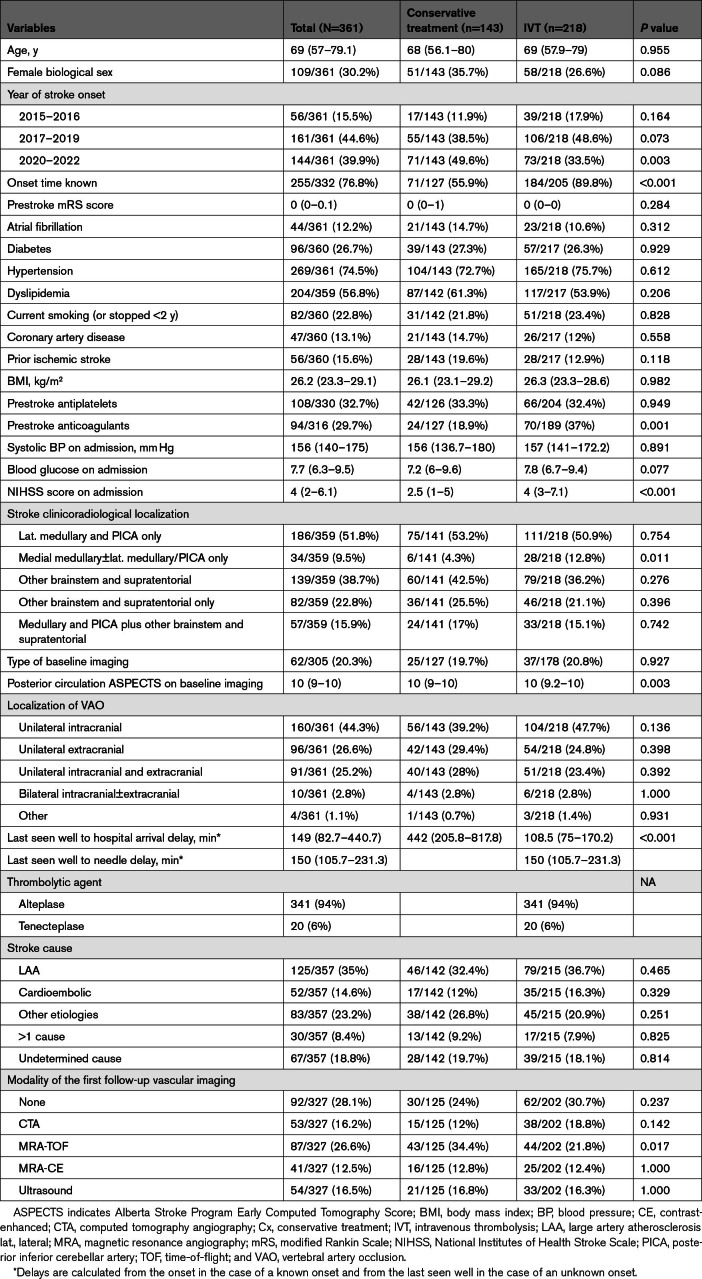
Baseline Characteristics of the Cx and IVT Groups

**Table 2.**
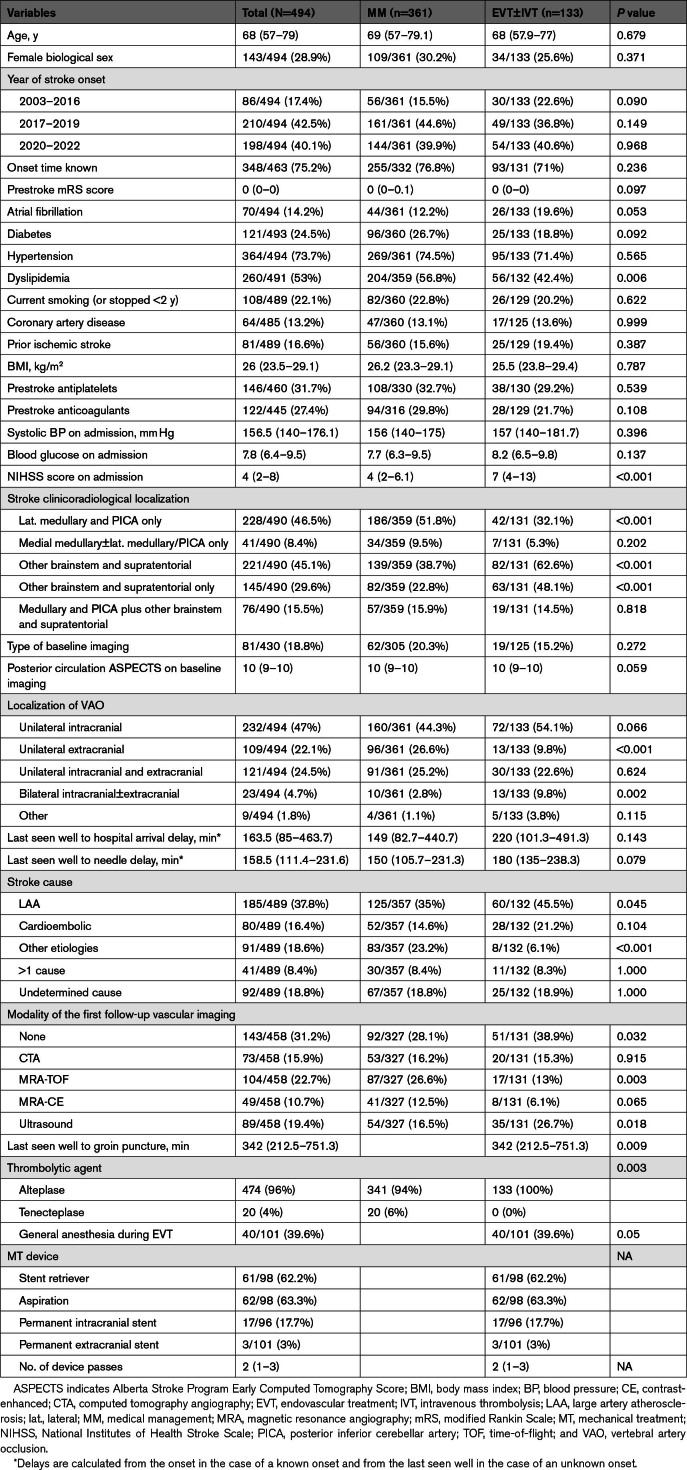
Baseline Characteristics of the MM and EVT±IVT Groups

Individuals receiving Cx therapy had a lower median admission NIHSS score than those receiving IVT-only (2.5 [IQR, 1–5] versus 4 [IQR, 3–7]; *P*<0.001). Individuals receiving MM had lower stroke severity in terms of admission NIHSS score than those receiving EVT±IVT (4 [IQR, 2–6.1] versus 7 [IQR, 4–13]; *P*<0.001). The EVT±IVT group more frequently had clinical-radiological lesions in the brainstem outside the VA territory and supratentorial lesions than the MM group. Isolated extracranial vertebral artery occlusions were less common in the EVT±IVT group.

#### IVT-Only Versus Cx

For the IVT-only versus Cx contrast analyses, the mRS score could be analyzed for 214 of 218 and 141 of 143 patients, respectively. Other secondary outcome data exploited for analyses are reported in Figure S1 and Table S6A through S6H.

Patients treated with IVT-only had slight differences in favor of IVT on mRS score distribution, good outcome, and excellent outcome, but those were not statistically significant in univariable and multivariable analyses (mRS shift score: IPTW-adjusted odds ratio [aOR], 1.32 [95% CI, 0.80–2.18]; Table 3). They showed greater NIHSS score improvement at 24 hours (multivariable regression analysis–adjusted β coefficient, −0.54 [95% CI, −1.07 to −0.01]; IPTW-adjusted-β coefficient, 1 [95% CI, 2.05–0.05]), and partial or full recanalization was significantly higher in the IVT group compared with Cx, with IVT increasing the odds of achieving a partial or full arterial recanalization on follow-up imaging by >4×(IPTW-aOR, 4.33 [95% CI, 1.36–13.78]; Table 3).

**Table 3.**
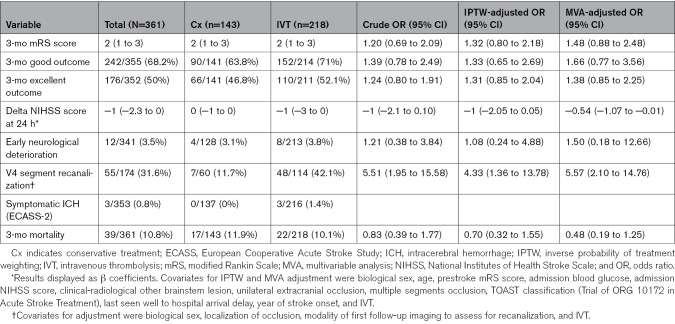
Functional and Safety Outcomes in the IVT Versus Cx Comparison

The rate of sICH according to the ECASS-2 definition and ENDi appeared not statistically different in the IVT and Cx groups (sICH: 1.4% versus 0%; ENDi: 3.8% versus 3.1%; IPTW-aOR, 1.08 [95% CI, 0.24–4.88]; Table 3).

The 3-month mortality was not statistically different in the IVT and Cx groups (10.1% versus 11.9%, IPTW-aOR, 0.70 [95% CI, 0.32–1.55]; Table 3).

#### EVT±IVT Versus MM

For the EVT±IVT versus MM contrast analyses, mRS score could be analyzed for 123 of 133 and 355 of 361 patients, respectively. Other secondary outcome available data exploited for analyses are reported in Figure S1 and Table S7A through S7H.

Patients treated with EVT±IVT had significantly worse mRS score distribution at 3 months (IPTW-aOR for favorable mRS shift score, 0.51 [95% CI, 0.35–0.74]), lower rates of good outcome (IPTW-aOR, 0.60 [95% CI, 0.36–1.00]; Figure S4), or significantly lower chances of excellent outcome (IPTW-aOR, 0.43 [95% CI, 0.26–0.72]; Figure S5). We did not observe any difference in the rate of 24-hour neurological improvement (IPTW-adjusted-β coefficient, 1.00 [95% CI, −0.28 to 2.28]; Table 4). ENDi occurred significantly more frequently in the EVT-treated population (25.4% versus 3.5%; IPTW-aOR, 9.06 [95% CI, 2.86–28.67]; Table 4).

**Table 4.**
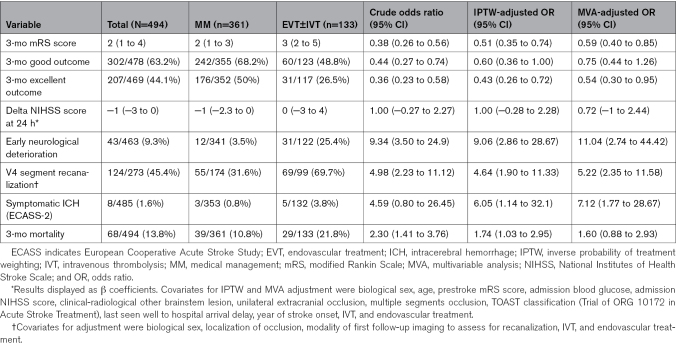
Functional and Safety Outcomes in the EVT±IVT Versus MM Comparison

Arterial recanalization at 24 hours was more frequent in EVT±IVT compared with MM, with EVT±IVT increasing the odds of achieving a partial or full arterial recanalization on follow-up imaging by >4×(IPTW-aOR, 4.64 [95% CI, 1.90–11.33]; Table 4).

EVT±IVT was associated with a statistically significant increase in sICH compared with MM (3.8% versus 0.8%; IPTW-aOR, 6.05 [95% CI, 1.14–32.1]). All sICHs corresponded to parenchymal hemorrhage, and no subarachnoid hemorrhage was observed as a postprocedural complication among our cohort of EVT-treated patients. Importantly, no patient who underwent permanent stent placement experienced intracerebral or subarachnoid bleeding.

Detailed characteristics and complications of the mechanical treatment procedure are reported in Tables S8 and S9, respectively. Among EVT±IVT–treated patients, acute permanent stent placement was performed in 20 of 95 procedures (21%), while general anesthesia was used in 40 of 101 (40%). Procedural complications occurred in 17 of 102 interventions (16.7%). Despite the higher rate of ENDi in the EVT±IVT group, none of the recorded periprocedural events was independently associated with an increased likelihood of ENDi (odds ratio, 1.23 [95% CI, 0.39–3.92]), and the use of general anesthesia was similarly not associated with ENDi (odds ratio, 0.46 (95% CI, 0.18–1.17)).

The 3-month mortality was higher in the EVT±IVT compared with MM (21.8% versus 10.8%; IPTW-aOR, 1.74 [95% CI, 1.03–2.95]; Table 4; Figure S6).

**Figure 1.**
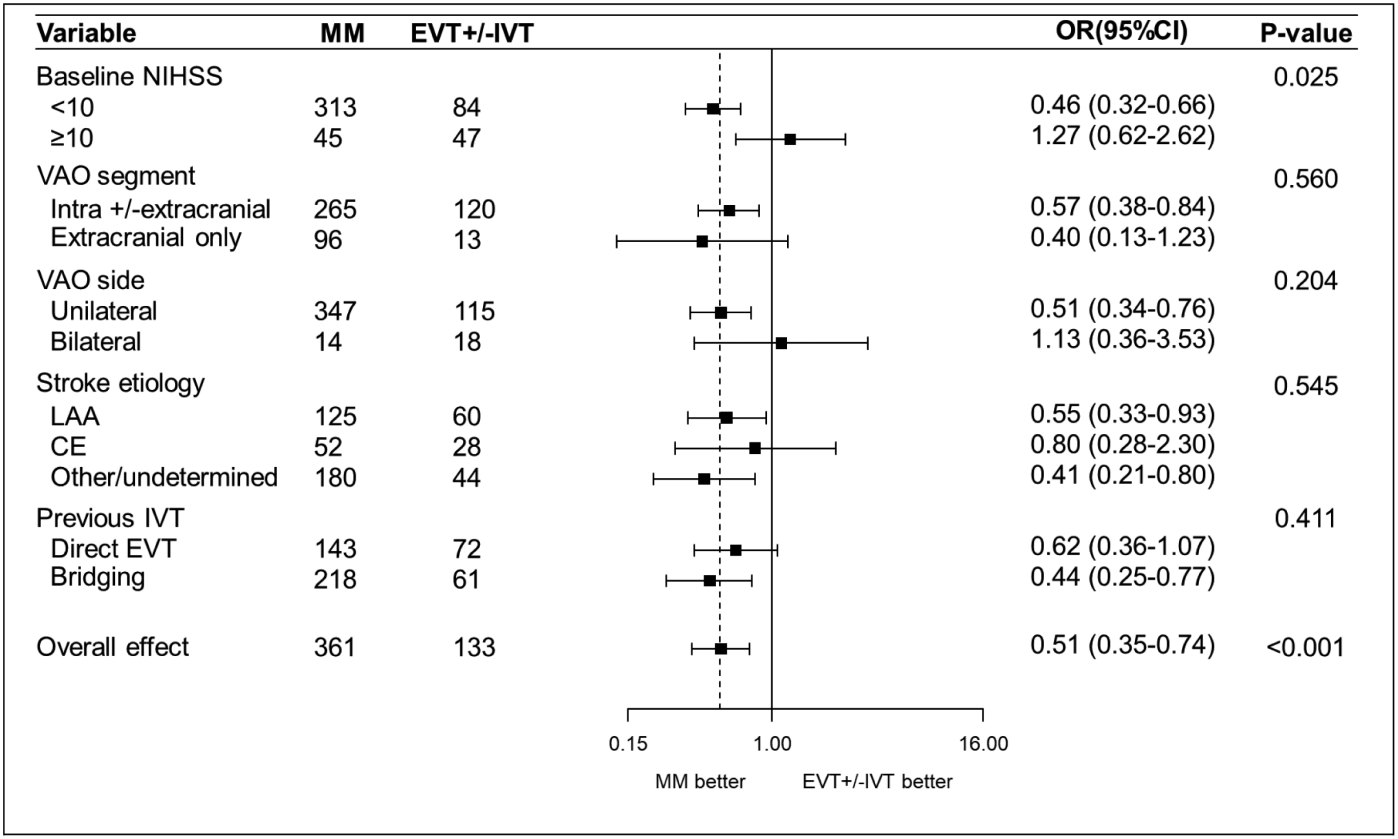
**Subgroup analysis.** Comparison of the occurrence of the primary outcome (modified Rankin Scale score, 0–2) between endovascular treatment and medical management groups according to key subgroups. Dotted line: overall effect of endovascular treatment (EVT)±intravenous thrombolysis (IVT) vs medical management (MM). CE indicates cardioembolic; LAA, large artery atherosclerosis; NIHSS, National Institutes of Health Stroke Scale; OR, odds ratio; and VAO, vertebral artery occlusion.

**Figure 2.**
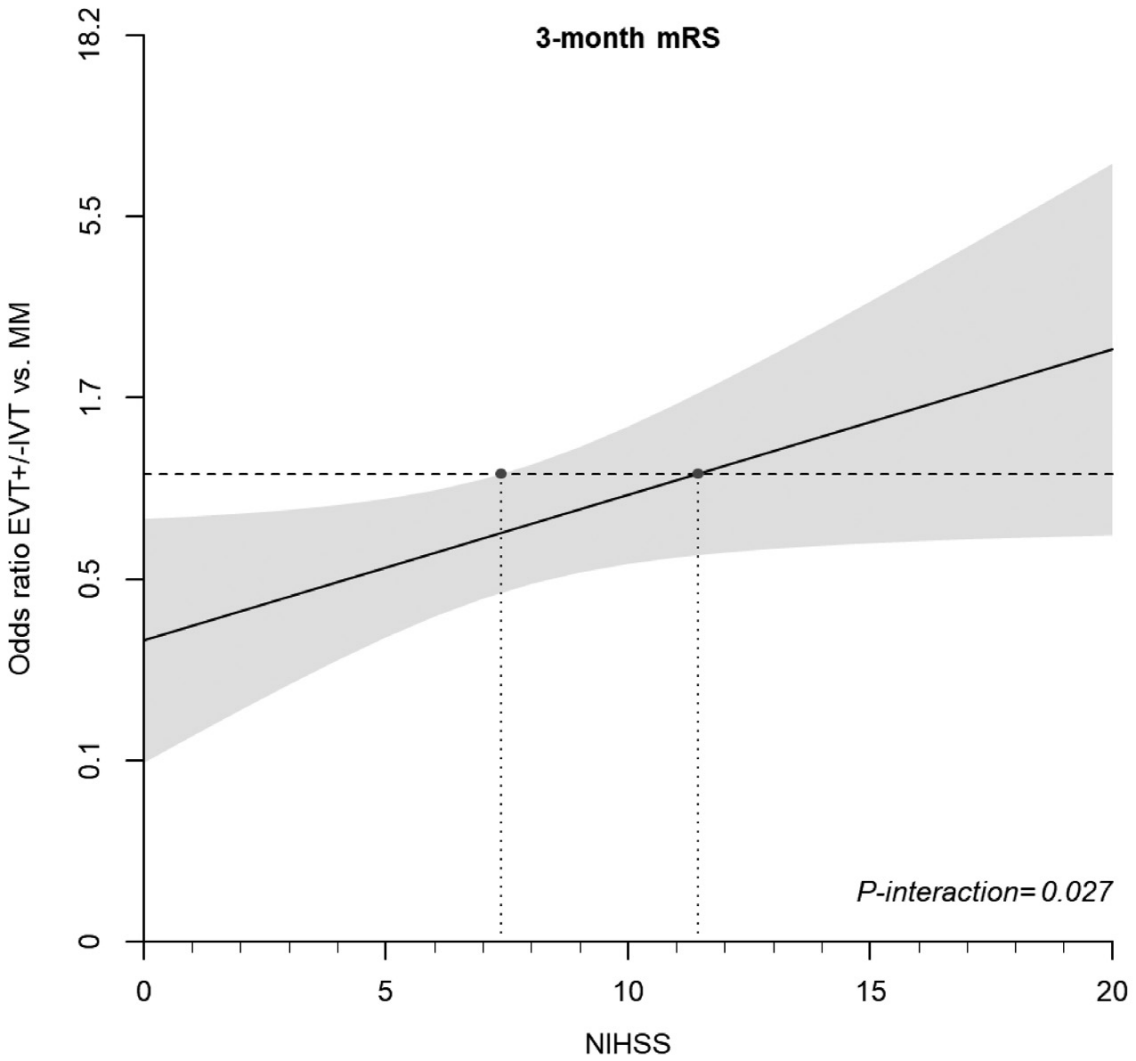
**Severity-outcome analysis.** Plot of odds ratio for favorable modified Rankin Scale (mRS) score toward admission National Institutes of Health Stroke Scale (NIHSS) score according to treatment in the endovascular treatment (EVT)±intravenous thrombolysis (IVT) vs medical management (MM) analysis.

Further subgroup analyses are presented in Figures 1 and 2. Specifically, when comparing EVT±IVT versus MM in individuals with admission NIHSS score ≥10, iVAO individuals seemed to fare better with EVT±IVT than MM (odds ratio, 1.27 [95% CI, 0.62–2.62]; *P*_interaction_=0.025).

## Discussion

This first large international observational study on individuals with AIS and iVAO unveils compelling divergence in the associations of acute revascularization therapies with functional outcomes. Notably, IVT-only compared with Cx shows similar safety and functional outcomes despite a higher frequency of neurological improvement and recanalization rate, while EVT±IVT exhibits overall lower odds of achieving favorable results compared with MM.

### Relationship Between IVT and Outcome

**Figure 3.**
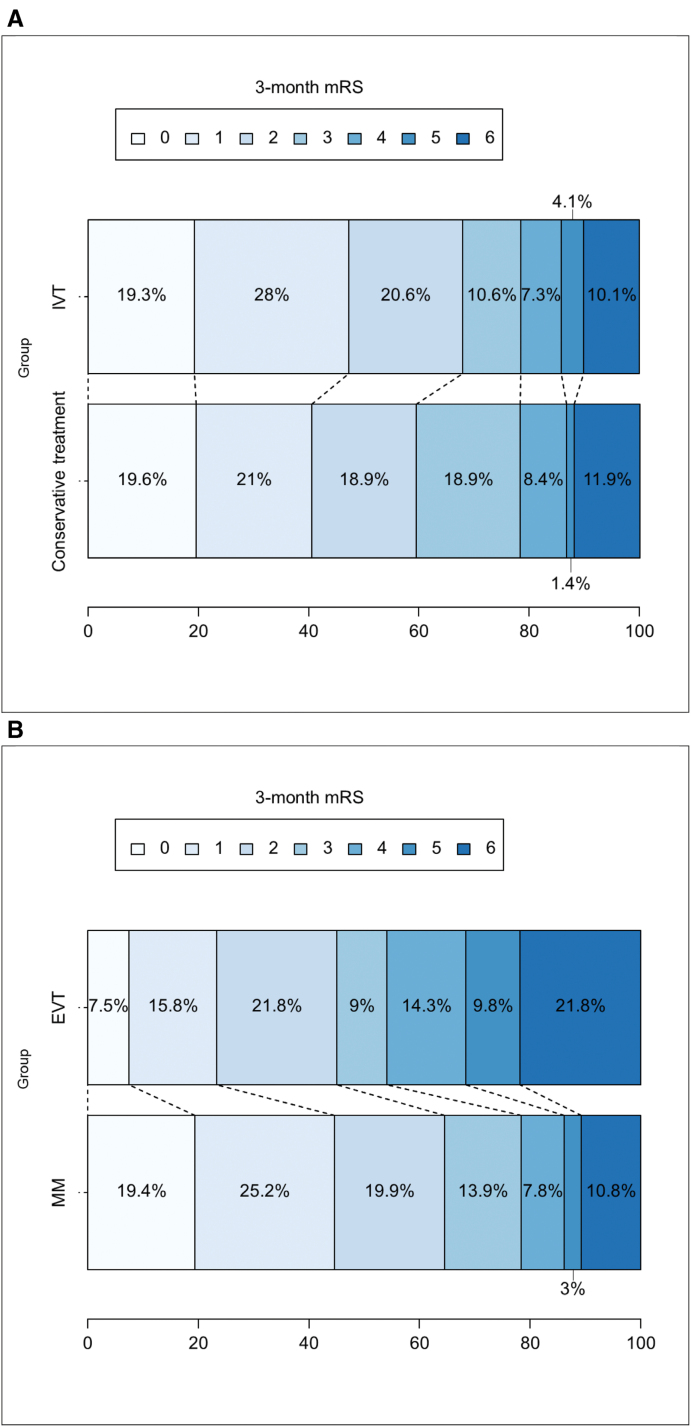
**Modified Rankin Scale (mRS) score distribution at 90-day across comparison groups. A**, The mRS score distribution at 90-day in the conservative treatment vs intravenous thrombolysis (IVT). Grotta bars showing mRS score distribution at 90 days in the conservative treatment group vs the IVT group. **B**, mRS score distribution at 90-day in the medical management (MM) vs endovascular treatment (EVT)±IVT. Grotta bars showing mRS score distribution at 90 days in the MM group vs EVT±IVT group.

Regarding the effect of IVT, we found no published data specifically focused on the iVAO population. However, post hoc analyses of the IST-3 trial (The Third International Stroke Trial)^8^ suggest overall effectiveness of IVT in PC ischemic strokes regardless of the presence of an occlusion. In addition, retrospective analyses indicate that IVT might be associated with better outcomes in the PC compared with the anterior circulation.^6^ While we could not demonstrate a significant association of IVT with a better 3-month mRS score, our findings on early neurological improvement, arterial recanalization, and safety outcomes corroborate the positive effect of IVT (Figure 3A; Table 3). Of note, we found that IVT was associated with an over 4× significantly higher likelihood of partial or complete arterial recanalization. The net difference in recanalization rates was 28.3% compared with the Cx group. While RCTs on IVT in general did not assess occlusion and recanalization status, our finding corroborates other retrospective studies investigating recanalization. Overall, IVT has been shown to increase recanalization rates of PC occlusive strokes by ≈22% (spontaneous=24.1%; IVT=46.2%),^23^ and in individuals with PCA occlusions, a significant 10× higher chance of recanalization with IVT compared with Cx was found with a net 42.4% increase in intracranial recanalization for IVT-treated individuals (Cx=9.1%; IVT=51.5%).^24^ The observation that recanalization after IVT is a robust predictor of good functional outcomes^23,25,26^ and IVT seems particularly associated with higher odds of achieving better outcomes in individuals with documented occlusions^27^ and further supports its use in iVAO.

Our study yielded no statistically significant difference in the rate of sICH between IVT and Cx, as per the ECASS-2 definition. These safety findings are consistent with IVT implementation studies in PC strokes, which demonstrated lower bleeding rates in PC compared with anterior circulation strokes.^6,9^ In addition, we found a 3.8% risk of ENDi in IVT-treated individuals, which is consistent with the 5% rate observed in the literature in all IVT-treated PC strokes, regardless of the presence of occlusion.^28^

### Relationship Between EVT±IVT and Outcome

We observed significantly less favorable outcomes at 3 months with EVT±IVT compared with MM (Figure 3B; Table 4). This finding was shown in both the propensity-weighted analysis and the adjusted ordinal regression analysis and is similar to the subgroup analysis of 12 individuals with dominant iVAO in the BEST study.^17^ Such results were obtained from individuals admitted after 2015, corresponding to the introduction of stent retrievers in routine clinical practice and general improvement of thrombectomy techniques. A possible explanation lies in the increased number of both sICH and ENDi in the EVT±IVT group, which may neutralize the positive effect of EVT±IVT in terms of arterial recanalization (69.7% versus 31.6% in the MM group). Interestingly, the higher number of sICH, ENDi, and 3-month mortality in the BRAVO cohort is not explained by a higher rate of procedural complication, which are similar to those seen in the general stroke population^29^ (17/102, 16.6% in BRAVO versus 16% in general stroke population) and which were not associated to a higher likelihood of having an ENDi, indicating that technical feasibility is not a limiting factor. In our cohort, no patient who underwent permanent stent placement, therefore likely to receive dual-antiplatelet therapy, experienced either intracerebral or subarachnoid hemorrhage. This suggests that, in BRAVO, the hemorrhagic events observed after EVT were not related to stent placement or its antiplatelet management.

Furthermore, 3-month mortality in our EVT group was similar to that reported by previous studies on the PC (29/133, 21.8% for EVT±IVT in BRAVO, compared with 24%–45% in EVT±IVT reported in the literature for any PC occlusion and BAO meta-analysis).^30,31^ Finally, this finding differs from the results of recent RCTs on BAO, in which EVT proved to be effective despite rates of sICH and ENDi like those observed in our EVT-treated group.^32,33^ In the only available retrospective study analyzing individuals with combined VAO and BAO, functional outcomes did not differ significantly between EVT±IVT versus IVT alone. While differences in technique across centers cannot be entirely excluded, these data do not indicate a predominant reliance on any single method. Nonetheless, there was a numerical increase in bleeding complications in the case of EVT+IVT.^30^ The Spearman correlation analysis between ENDi severity described in terms of NIHSS score point degradation and 3-month disability within the EVT subgroup showed a moderate positive association (Figure S7; ρ=0.345; *P*<0.001), indicating that greater early neurological deterioration was significantly associated with worse functional outcomes at 3 months.

These findings point to the complex interplay between baseline clinical severity, reperfusion timing, and procedural risk, which may partly explain why endovascular therapy in iVAO has not replicated the clear functional benefit observed in anterior circulation strokes or BAOs. In reviewing the literature of other PC segments, the impact of EVT appears to be heterogeneous. For instance, in individuals with BAO, factors such as clinical stroke severity and thrombolysis administration could potentially modify the magnitude of EVT benefit within the same trial population or explain the heterogeneous results of BASICS (Basilar Artery International Cooperation Study)^34^ and BEST^17^ compared with ATTENTION (Endovascular Treatment for Acute Basilar Artery Occlusion)^12^ and BAOCHE (Basilar Artery Occlusion Chinese Endovascular Trial).^13^ Similarly, in PCA occlusions, diverse clinical outcomes have been reported in different retrospective comparisons.^24,35–38^

A direct comparison between EVT±IVT and IVT-only was considered but not performed, given the major temporal and clinical imbalances between these subgroups and the risk of additional selection bias.

Furthermore, vertebral artery dominance represents a critical anatomic determinant of hemodynamic compensation and clinical severity in PC stroke. In cases of iVAO, involvement of the dominant vertebral artery may produce a hemodynamic pattern more closely resembling proximal BAO. This concept was reflected in the BAOCHE randomized trial, which treated bilateral iVAO as a surrogate for proximal BAO.^13^ Future prospective or randomized controlled studies should specifically focus on symptomatic, imaging-confirmed vertebral artery occlusions and incorporate standardized assessments of hemodynamics and vertebral artery dominance to reduce heterogeneity and enhance comparability across centers.

Our subgroup analysis examining the patients with moderate-to-severe clinical manifestations of stroke, consisting of an admission NIHSS score ≥10, also showed higher odds of better outcome, favoring EVT (Figures 1 and 2). Nonetheless, it must be underlined that the standard NIHSS score underestimates PC symptoms and may not, therefore, be the optimal way to quantify deficit in vertebral artery occlusion. Further prospective or randomized controlled studies should investigate the use of the POST-NIHSS score.^4^ This scale may help to establish a more reliable threshold to assess the tradeoff between risk and benefit of endovascular procedures.

Results from the subgroup analyses, therefore, support the hypotheses that clinically (admission NIHSS score ≥10) moderate-to-severe strokes might still benefit from EVT. Such findings seem to be in line with the most recent results of BAO trials.

### Limitations

The retrospective design may be susceptible to multiple biases inherent to its retrospective, multicenter, observational design, which we addressed with measures to overcome bias reported in Table S10. Although we applied IPTW using a machine learning–based generalized boosted model to minimize selection bias, residual confounding by unmeasured variables cannot be excluded. Balance diagnostics (Figure S2A through S2D) showed a clear improvement in covariate distribution postweighting, and most variables reached acceptable standardized mean differences. Nonetheless, a limited number of covariates retained an absolute standardized mean difference >10%, indicating some minor residual imbalances. These differences were considered unlikely to meaningfully alter the direction of the results but underscore that the analyses remain associative. The heterogeneity of clinical presentations, imaging patterns, and endovascular techniques across centers represents an intrinsic feature of real-world, international data collection. Accordingly, the associations reported here should be interpreted with caution and not as evidence of causality. The analyses were designed to describe treatment patterns, outcomes, and safety profiles rather than to establish therapeutic superiority. Not all centers contributed patients to each treatment category (Table S3), reflecting differences in treatment availability and local practice; cluster-robust modeling was, therefore, used to account for site-level variability. The study’s power to detect differences might be low for low-frequency outcomes such as those of sICH in the Cx versus IVT-only comparison. In these situations, results should be interpreted with caution. Also, our cohort included individuals mainly from endovascular-capable centers, and this could, therefore, influence representativeness or generalizability with respect to the broader population. The nonblinded assessment may have led to reporting bias. We also acknowledge that the determination of vertebral artery dominance was not feasible in this study, as imaging data were collected retrospectively across multiple centers without centralized review. In some cases, it might also be difficult to clearly distinguish between an acute bilateral vertebral artery occlusion and a combination of an acute unilateral occlusion with a contralateral severely hypoplastic or chronically occluded artery based on initial admission imaging alone. These limitations reflect the challenges of assessing vertebral artery anatomy and flow contribution and underscore the importance of future multicenter efforts with harmonized centralized imaging review and adjudication to ensure consistent evaluation of vertebral artery dominance and collateral hemodynamics. We have not recorded second intention revascularization procedures, for example, for clinical worsening after admission. The exact timing of early neurological deterioration relative to EVT could not be determined, as the cohort study did not systematically capture the temporal sequence of events within the first 24 hours. This precluded distinguishing between neurological decline occurring before, versus after, the procedure, a question that warrants prospective evaluation in future studies. Finally, some of the subgroup analyses were constrained by missing data, which we have fully reported in Table S5.

### Conclusions

In conclusion, our study confirms that despite representing almost 20% of all PC occlusions, iVAO has been a neglected stroke syndrome in the setting of revascularization treatments. Our results, along with the collective body of knowledge derived from general stroke management findings and studies focusing on other PC arterial segments, contribute to supporting the use of IVT as the treatment option of choice for the management of AIS related to iVAO. On the contrary, our findings indicate lower odds of a favorable outcome for EVT±IVT–treated individuals compared with MM, possibly related to higher ENDi and sICH rates. Nonetheless, subgroup analyses of moderate-to-severe strokes and bilateral iVAO show point estimates in favor of EVT for the management of such cases.

Given the absence of dedicated RCTs and the heterogeneous outcomes observed in other PC segments, additional research is warranted to optimize the management of symptomatic iVAO strokes. There is a need for further prospectively collected iVAO registries or even RCTs to assess if EVT might be of added value in addition to MM.

## ARTICLE INFORMATION

### Acknowledgments

The authors would like to thank Dr Marie-Annick Le Pogam, MD, MPH, PhD, and Dr Mohamed Faouzi, PhD, for their support in the revision of the protocol of the study and Dr Melanie Price Hirt, PhD, for English language correction and editing.

### Author Contributions

Drs Salerno and Strambo were involved in conceptualization, data curation, formal analysis, methodology, original draft, and writing-review & editing. Dr Michel was involved in conceptualization, data curation, formal analysis, methodology, supervision, original draft, and writing-review & editing. All other authors were involved in data acquisition and curation, and writing-review & editing.

### Sources of Funding

### Disclosures

Drs Salerno, Strambo, and Michel report grants from the University of Lausanne. Dr Padjen reports travel support from Boehringer Ingelheim and grants from Pfizer Canada Inc, and Medtronic. Dr Heldner reports grants from the Swiss Heart Foundation, the SITEM Research Funds, the Swiss National Science Foundation, and the Gottfried und Julia Bangerter-Rhyner-Stiftung. Dr Nolte reports compensation from Bayer Healthcare, Bristol Myers Squibb, Alexion Pharmaceuticals, AstraZeneca, and Pfizer for consultant services. Dr Zini reports compensation from Amgen, Daiichi Sankyo, Alexion Pharmaceuticals, CSL Behring, Pfizer, and Boehringer Ingelheim for consultant services; is a principal investigator for TAILOR trial (Tailored Antiplatelet Secondary Prevention in Non-Cardioembolic Ischemic Stroke: A Phase IV Gender-Stratified Randomized Controlled Trial); EU CT Number: 2025-523065-16-00 for principal investigator for trial TAILOR; reports compensation from Bayer Healthcare for other services; grants from Italian Health Ministry; and is a principal investigator for project RF-2019-12370834-FibER: Fibrinogen Replacement to Prevent Intracranial Haemorrhage in Ischemic Stroke Patients After Thrombolysis: A Pilot RCT (sponsored by Grant of the Italian Health Ministry), EudraCT 2020-005242-41 for principal investigator for project RF-2019-12370834-RCT FibER ClinicalTrials.gov: NCT05300672. Dr Nguyen reports compensation from Genentech, the American Stroke Association, and Kaneka for other services and compensation from Brainomix, Route 92 Medical Inc, Medtronic, and Aruna for consultant services. Dr Gensicke reports grants from Daiichi Sankyo Company, Ipsen, AbbVie, and Merz Pharma (Schweiz) AG, and travel support from AbbVie. Dr Kellert reports compensation from Boehringer Ingelheim, Pfizer, AstraZeneca, Daiichi Sankyo, Bayer, Bristol Myers Squibb, Eli Lilly, and Alexion Pharmaceuticals for other services. Dr Siegler reports compensation from AstraZeneca for other services; employment by the University of Chicago; grants from viz.ai, Medtronic, and Philips; and compensation from Novartis for consultant services. Dr Machi reports compensation from MicroVention Inc, Medtronic, Penumbra Inc, and Stryker for consultant services. Dr Fiehler reports compensation from Medtronic USA Inc, Medical, MicroVention Inc, Roche, Phenox, Cerenovus, Penumbra Inc, Stryker Corporation, Acandis, and Tonbridge for consultant services; stock holdings in Eppdata, Tegus Medical, and Vastrax; and employment by Eppdata. Dr Leker reports compensation from Bayer, Abbott Diabetes Care, Medtronic, Ischemaview, Biogen, Janssen Biotech, and Boehringer Ingelheim for other services; grants from Horizon 2020 Framework program; and compensation from Filterlex for consultant services. Dr Cereda reports compensation from Medtronic, AstraZeneca, and Bayer for consultant services, and compensation from iSchemaView for other services. Dr Cordonnier reports compensation from Bayer for consultant services; compensation from Biogen, Bayer, the American Stroke Association, and Boehringer Ingelheim for other services; and employment by the University of Lille. Dr Engelter reports grants from Daiichi Sankyo and travel support from Pfizer, Boehringer Ingelheim, and Bayer. The other authors report no conflicts.

### Supplemental Material

Tables S1–S10

Figures S1–S7

STROBE Checklist

## Supplementary Material



## Appendix

### EVA-TRISP Investigators (Co-Authors)

Susanne Wegener, MD; Stefania Maffei, PhD; Livio Picchetto, MD; Kateryna Antonenko, MD; Marcel Arnold, MD; Christoph Riegler, MD; Jan Friedrich Scheitz, MD; Mauro Gentile, MD; Simon Truessel, BSc; Johannes Wischmann, MD; Annika Nordanstig, MD, PhD; Luca Frediani, MD; Laura Giacobazzi, MD; Pasquale Mordasini, MD; Jan Gralla, MD; Ludovica Migliaccio, BSc; Giovanni Bianco, MD; François Caparros, MD; Hilde Henon, MD, PhD; Rosario Pascarella, MD; Manuela Napoli, MD; Ilaria Grisendi, MD; Fanny Simonnet, MScNP

BRAVO Investigators (Co-Authors)

Brian Mac Grory, MD; Soo Jeong, MD; Mohamad Abdalkader, MD; Mohammad J. Ahmad; MBBS; José Bernardo Escribano Paredes, MD; Fabian Flottmann, MD; Julia Ferrari, MD; Michael Knoflach, MD; Federica Rizzo, MD; Ahmad Zamzam, MD; Parth Patel, MD; Srishti Dhar, BSC; Judith Clark, BSc; Muhammad Bilal Tariq, MBBS; Emmanuel Carrera, MD; Marc Ribo, MD; Lars-Peder Pallesen, MD
