# Supplementary material for: Outcomes and Safety of Revascularization Approaches for Stroke Related to Isolated Vertebral Artery Occlusions (BRAVO)
Source: Stroke. 2026 Mar 10;57(5):1149–63. doi: 10.1161/STROKEAHA.125.051675 (PMC13117616; doi:10.1161/STROKEAHA.125.051675)

Outcomes and Safety of Revascularization Approaches for Stroke Related to Isolated Vertebral Artery Occlusions (BRAVO)

SUPPLEMENTAL MATERIAL

Alexander Salerno, MD,PhD et al.

Table of Contents

Table S1: STROBE Statement – Checklist of items that should be included in reports of observational studies 2

Table S2: Investigators of the EVA-TRISP and BRAVO consortia 5

Table S3: Participating institutions and analyzed provided data 7

Table S4: Collected variables 9

Table S5: Missing data of the included patients (N=494) 13

Table S6: Assessment for risk of bias 15

Table S7: Detailed statistical analysis for IVT vs Cx groups 16

Table S8: Detailed statistical analysis for EVT±IVT vs MM groups 24

Table S9: Detailed characteristics of mechanical treatment procedure 32

Table S10: Detailed procedural complications of mechanical treatment 33

Figure S1: Exclusion flowchart 34

Figure S2: Severity-Outcome analysis for EVT vs MM good outcome 35

Figure S3: Severity-Outcome analysis for EVT vs MM excellent outcome 36

Figure S4: Severity-Outcome analysis for EVT vs MM mortality 37

Figure S5: Spearman correlation between ENDi severity described in terms of NIHSS point degradation and 3-month disability within the EVT subgroup 38

Figure S6: Standardized mean difference (SMD) for multiple imputed datasets (Unweighted vs. Weighted) 39

Figure S7: Propensity score distribution in the models 43

Table S1: STROBE Statement – Checklist of items that should be included in reports of observational studies

	Item No.	Recommendation	Page No.	Relevant text from manuscript
**Title and abstract**	1	(*a*) Indicate the study's design with a commonly used term in the title or the abstract	1,3	Title, Abstract
		(*b*) Provide in the abstract an informative and balanced summary of what was done and what was found	3,4	Methods and Findings paragraphs of the abstract
**Introduction**				
Background/rationale	2	Explain the scientific background and rationale for the investigation being reported	5-6	Background section
Objectives	3	State specific objectives, including any prespecified hypotheses	6	Last paragraph of the background section
**Methods**				
Study design	4	Present key elements of study design early in the paper	6	Study design chapter of the methods section
Setting	5	Describe the setting, locations, and relevant dates, including periods of recruitment, exposure, follow-up, and data collection	6,7	Population chapter of the methods section
Participants	6	(*a*) *Cohort study*—Give the eligibility criteria, and the sources and methods of selection of participants. Describe methods of follow-up	6,7	Population chapter of the methods section
		(*b*) *Cohort study*—For matched studies, give matching criteria and number of exposed and unexposed	N/A	Not applicable, propensity-weighting method applied
Variables	7	Clearly define all outcomes, exposures, predictors, potential confounders, and effect modifiers. Give diagnostic criteria, if applicable	7,8	Treatments and Measures chapters of the methods section
Data sources/ measurement	8*	For each variable of interest, give sources of data and details of methods of assessment (measurement). Describe comparability of assessment methods if there is more than one group	8, Supplementary Appendix	Measures chapter of the methods section; Specific measures in the Supplementary Appendix
Bias	9	Describe any efforts to address potential sources of bias	Supplementary Appendix	Risk of bias assessment table in Supplementary Appendix
Study size	10	Explain how the study size was arrived at	6,7	Population chapter in the methods section

Continued on next page

Quantitative variables	11	Explain how quantitative variables were handled in the analyses. If applicable, describe which groupings were chosen and why	8-10	Statistical analysis chapter in methods section
Statistical methods	12	(*a*) Describe all statistical methods, including those used to control for confounding	8-10	Statistical analysis chapter in methods section
		(*b*) Describe any methods used to examine subgroups and interactions	8-10	Statistical analysis chapter in methods section
		(*c*) Explain how missing data were addressed	8-10, Supplementary Appendix	Statistical analysis chapter in the methods section; Missing data table in Supplementary Appendix
		(*d*) *Cohort study*—If applicable, explain how loss to follow-up was addressed	Supplementary Appendix	Risk of bias assessment table in Supplementary Appendix
		(*e*) Describe any sensitivity analyses	Supplementary Appendix	Complete case analysis table in Supplementary Appendix
**Results**				
Participants	13*	(a) Report numbers of individuals at each stage of study—eg numbers potentially eligible, examined for eligibility, confirmed eligible, included in the study, completing follow-up, and analysed	11, Supplementary Appendix	Population characteristics chapter in the results section, Exclusion flowchart in the Supplementary Appendix
		(b) Give reasons for non-participation at each stage	Supplementary Appendix	Exclusion flowchart in the Supplementary Appendix
		(c) Consider use of a flow diagram	Supplementary Appendix	Exclusion flowchart in the Supplementary Appendix
Descriptive data	14*	(a) Give characteristics of study participants (eg demographic, clinical, social) and information on exposures and potential confounders	11	Population characteristics chapter in the results section
		(b) Indicate number of participants with missing data for each variable of interest	Supplementary Appendix	Missing data table in Supplementary Appendix
		(c) *Cohort study*—Summarise follow-up time (eg, average and total amount)	8	Measures chapter in the methods section
Outcome data	15*	Cohort study—Report numbers of outcome events or summary measures over time	11-12	Primary effectiveness outcomes, Secondary outcomes and Safety outcomes chapter in the results section
Main results	16	(*a*) Give unadjusted estimates and, if applicable, confounder-adjusted estimates and their precision (eg, 95% confidence interval). Make clear which confounders were adjusted for and why they were included	11-12	Primary effectiveness outcomes, Secondary outcomes and Safety outcomes chapter in the results section

		(*b*) Report category boundaries when continuous variables were categorized	11-12	Subgroup analysis chapter in the results section
		(*c*) If relevant, consider translating estimates of relative risk into absolute risk for a meaningful time period	N/A	Not applicable since not randomized clinical data
Other analyses	17	Report other analyses done—eg analyses of subgroups and interactions, and sensitivity analyses	11-12, Supplementary Appendix	Subgroup analysis chapter in the results section, Complete case analysis table in Supplementary Appendix
**Discussion**				
Key results	18	Summarise key results with reference to study objectives	13-16	Relationship between IVT and outcome and relationship between EVT and outcome chapters in the discussion section
Limitations	19	Discuss limitations of the study, taking into account sources of potential bias or imprecision. Discuss both direction and magnitude of any potential bias	16	Strengths and limitations chapter of the discussion section
Interpretation	20	Give a cautious overall interpretation of results considering objectives, limitations, multiplicity of analyses, results from similar studies, and other relevant evidence	13-17	Relationship between IVT and outcome and relationship between EVT and outcome chapters in the discussion section, conclusions section
Generalisability	21	Discuss the generalisability (external validity) of the study results	16-17	Relationship between IVT and outcome and relationship between EVT and outcome chapters in the discussion section
**Other information**				
Funding	22	Give the source of funding and the role of the funders for the present study and, if applicable, for the original study on which the present article is based	10	Role of the funding source chapter in the methods section

*Give information separately for cases and controls in case-control studies and, if applicable, for exposed and unexposed groups in cohort and cross-sectional studies.

Table S2: Investigators of the EVA-TRISP and BRAVO consortia

**EVA-TRISP Investigators (co-authors)**			
Susanne Wegener	MD	0000-0003-4369-7023	Department of Neurology, University Hospital Zurich and University of Zurich
Stefania Maffei	PhD	0000-0002-6428-4300	Stroke Unit, Neurology Clinic, Ospedale Civile di Baggiovara, Azienda Ospedaliero-Universitaria di Modena, Italy
Livio Picchetto	MD	0000-0001-6684-8117	Stroke Unit, Neurology Clinic, Ospedale Civile di Baggiovara, Azienda Ospedaliero-Universitaria di Modena, Italy
Kateryna Antonenko	MD	0000-0002-1936-5451	Stroke Unit, Department of Neurology, Inselspital, Bern University Hospital, University of Bern, Bern, Switzerland
Marcel Arnold	MD	0000-0002-4274-4644	Stroke Unit, Department of Neurology, Inselspital, Bern University Hospital, University of Bern, Bern, Switzerland
Christoph Riegler	MD	0000-0002-2478-3500	Department of Neurology, Charité - Universitätsmedizin Berlin, Corporate member of Freie Universität Berlin and Humboldt Universität zu Berlin, Berlin, Germany
Jan Friedrich Scheitz	MD	0000-0001-5835-4627	Department of Neurology, Charité - Universitätsmedizin Berlin, Corporate member of Freie Universität Berlin and Humboldt Universität zu Berlin, Berlin, Germany
Mauro Gentile	MD	0000-0002-6912-8868	IRCCS Istituto delle Scienze Neurologiche di Bologna, Department of Neurology and Stroke Center, Maggiore Hospital, Bologna, Italy
Simon Truessel	BSc		Neurology and Neurorehabilitation, University Department of Geriatric Medicine FELIX PLATTER, University of Basel, Switzerland
Johannes Wischmann	MD	0000-0003-0653-943X	Ludwig-Maximilians-Universitat Munchen; Munchen, Bayern, Germany
Annika Nordanstig	MD,PhD		Department of Neurology, Sahlgrenska University Hospital, Göteborg, Sweden
Luca Frediani	MD		Stroke Center EOC, Neurocenter of Southern Switzerland, Ospedale Regionale di Lugano, Ente Ospedaliero Cantonale, Lugano
Laura Giacobazzi	MD	0000-0002-6516-8002	Stroke Unit, Neurology Clinic, Ospedale Civile di Baggiovara, Azienda Ospedaliero-Universitaria di Modena, Italy
Pasquale Mordasini	MD	0000-0003-1712-4168	Institute for Diagnostic and Interventional Neuroradiology, Inselspital, Bern University Hospital, and University of Bern, Bern, Switzerland
Jan Gralla	MD	0000-0003-1953-9033	Institute for Diagnostic and Interventional Neuroradiology, Inselspital, Bern University Hospital, and University of Bern, Bern, Switzerland
Ludovica Migliaccio	BSc	0000-0001-7110-4892	IRCCS Istituto delle Scienze Neurologiche di Bologna, Department of Neurology and Stroke Center, Maggiore Hospital, Bologna, Italy
Giovanni Bianco	MD		Stroke Center EOC, Neurocenter of Southern Switzerland, Ospedale Regionale di Lugano, Ente Ospedaliero Cantonale, Lugano
François Caparros	MD	0000-0002-6627-0893	Univ. Lille, Inserm, CHU Lille Lille Neuroscience & Cognition, Lille, France
Hilde Henon	MD,PhD	0000-0001-6274-7562	Univ. Lille, Inserm, CHU Lille Lille Neuroscience & Cognition, Lille, France
Rosario Pascarella	MD	0000-0002-0512-9298	Neuroradiology Unit, Azienda Unità Sanitaria Locale-IRCCS di Reggio Emilia, Reggio Emilia, Italy
Manuela Napoli	MD	0000-0002-0434-4172	Neuroradiology Unit, Azienda Unità Sanitaria Locale-IRCCS di Reggio Emilia, Reggio Emilia, Italy
Ilaria Grisendi	MD	0000-0002-2939-6710	Neurology Unit, Stroke Unit, Azienda Unità Snaitaria Locale-IRCCS di Reggio Emilia, Reggio Emilia, Italy
Fanny Simonnet	MScNP	0009-0004-4458-6346	Stroke Center, Neurology Service, Centre Hospitalier Universitaire Vaudois, Switzerland

The BRAVO Observational, Multicenter, Cohort study

**BRAVO Investigators (co-authors)**			
Brian Mac Grory	MD	0000-0003-3914-8419	Department of Neurology, Duke University School of Medicine, Durham, NC, 27512, USA
Soo Jeong	MD		Department of Neurology, Asan Medical Center, Seoul, Korea
Mohamad Abdalkader	MD	0000-0002-9528-301X	Boston Medical Center, Boston University School of Medicine, Boston, MA, United States
Mohammad J Ahmad	MBBS	0009-0005-4972-5015	Department of Neurology, University of Texas McGovern Medical School, Houston, Texas, USA
José Bernardo Escribano Paredes	MD	0000-0003-0534-7962	Department of Clinical Neurosciences, Geneva University Hospital, Geneva, Switzerland
Fabian Flottmann	MD	0000-0001-8358-8089	Department of Diagnostic and Interventional Neuroradiology, University Medical Center Hamburg-Eppendorf, Hamburg, Germany
Julia Ferrari	MD	0000-0001-9709-0097	Department of Neurology, St. John's Hospital, Vienna, Austri
Michael Knoflach	MD	0000-0001-5576-6562	Department of Neurology, Medical University of Innsbruck, Austria
Federica Rizzo	MD	0000-0002-5945-8222	Stroke Unit, Department of Neurology, Hospital Universitari Vall d'Hebron, Barcelona, Spain
Ahmad Zamzam	MD		Department of Neurology, Duke University School of Medicine, Durham, NC, 27512, USA
Parth Patel	MD		Department of Neurology, Cooper Neurological Institute, Cooper University Hospital, Camden, New Jersey, USA
Srishti Dhar	BSc		Department of Neurology, Cooper Neurological Institute, Cooper University Hospital, Camden, New Jersey, USA
Judith Clark	BSc		Boston Medical Center, Boston University School of Medicine, Boston, MA, United States
Muhammad Bilal Tariq	MBBS		Department of Neurology, University of Texas McGovern Medical School, Houston, Texas, USA
Emmanuel Carrera	MD	0000-0003-0045-5382	Department of Clinical Neurosciences, Geneva University Hospital, Geneva, Switzerland
Marc Ribo	MD	0000-0001-9242-043X	Stroke Unit, Department of Neurology, Hospital Universitari Vall d'Hebron, Barcelona, Spain
Lars-Peder Pallesen	MD	0000-0002-0084-4620	Department of Neurology, University Clinics Carl Gustav Carus, Dresden, Germany

Table S3: Participating institutions and analyzed provided data

Center	Individuals	Cx	IVT-only	IVT+EVT	EVT-only	Start year	End year	Incidence analysis
Asan Medical Center<br>*Seoul, South Korea*	12	0	7	1	4	2016	2020	no
Austrian Stroke Unit Registry<br>*Vienna, Austria*	31	16	13	1	1	2016	2022	no
St. John's Hospital<br>*Vienna, Austria*	10	3	5	1	1	2016	2022	yes
Medizinische Universität Innsbruck<br>*Innsbruck, Austria*	10	10	0	0	0	2021	2022	yes
Vall d'Hebron University Hospital<br>*Barcelona, Spain*	1	0	1	0	0	2019	2021	yes
University Hospital Basel<br>*Basel, Switzerland*	23	0	20	1	2	2016	2021	no
University Clinical Centre of Serbia<br>*Belgrade, Serbia*	30	3	15	1	11	2016	2022	no
Charité-Universitätsmedizin Berlin<br>*Berlin, Germany*	22	5	10	1	6	2016	2022	yes
University Hospital Bern<br>*Bern, Switzerland*	19	0	17	1	1	2016	2021	no
Maggiore Hospital<br>*Bologna, Italy*	26	3	12	3	8	2019	2022	no
Boston Medical Center<br>*Boston, United States*	10	9	1	0	0	2021	2022	yes
University Hospital of Brescia<br>*Brescia, Italy*	13	0	0	10	3	2016	2021	no
Cooper University Hospital<br>*Camden, United States*	13	10	2	0	1	2019	2021	yes
University Clinics Carl Gustav Carus<br>*Dresden, Germany*	1	0	1	0	0	2018	2018	no
Duke University School of Medicine<br>*Durham, United States*	21	16	3	0	2	2016	N/A, anonym	no
University Hospital Geneva<br>*Geneva, Switzerland*	14	6	8	0	0	2019	2021	yes
Hadassah-Hebrew University Medical Center<br>*Jerusalem, Israel*	7	0	6	1	0	2016	2018	no
Sahlgrenska University Hospital<br>*Göteborg, Sweden*	8	0	0	2	6	2016	2020	no
University Medical Center Hamburg-Eppendorf<br>*Hamburg, Germany*	7	0	0	4	3	2016	2021	no
Helsinki University Hospital<br>*Helsinki, Finland*	31	0	26	2	3	2016	2020	no
McGovern Medical School<br>*Houston, Texas*	16	5	4	3	4	2018	2021	yes
Lausanne University Hospital	43	15	17	6	5	2003	2021	yes

Lausanne, Switzerland								
Centre Hospitalier Universitaire Lille<br>*Lille, France*	3	0	2	0	1	2016	2021	no
Hospital de Egas Moniz<br>*Lisbon, Portugal*	9	4	4	0	1	2016	2022	yes
Neurocenter of Southern Switzerland<br>*Lugano, Switzerland*	4	0	2	0	1	2020	2021	no
Michigan State University<br>*Grand Rapids, United States*	6	0	4	0	2	2019	2021	yes
Azienda Ospedaliero-Universitaria di Modena<br>*Modena, Italy*	32	0	12	20	0	2016	2021	no
Ludwig-Maximilians-Universitat<br>*Munchen, Germany*	16	3	7	1	5	2016	2022	yes
IRCCS di Reggio Emilia<br>*Reggio Emilia, Italy*	9	0	8	1	0	2016	2020	no
University Hospital Zurich<br>*Zurich, Switzerland*	47	35	10	1	1	2016	2021	yes
**TOTAL**	**494**	143	218	61	72			

Number of participants is referred to those who were included in the analysis according to the exclusion flowchart provided in Supplementary material.

Table S4: Collected variables

Collected variables included information on demographics, cerebrovascular risk factors and pre-stroke treatment, time-metrics, clinical presentation at time of treatment, imaging and angiographic characteristics, territory of PC stroke (vertebral, distal, multilevel), extent of VAO (intracranial, extracranial, or both), presumed stroke aetiology, revascularisation treatment, EVT technical aspects and complications, recanalization status, cause of death, and outcome at 3 months. PC specific territories were defined using clinical and/or radiological information that was retrospectively reviewed from local investigators. Estimated time of stroke was the time of symptom onset or of last proof of good health if stroke was unwitnessed.

The full metadata list includes:

1. "Center"
2. "age in years number"
3. "gender; [1]= Male; [2]= Female"
4. "Year of stroke onset; yyyy "
5. "Stroke onset witnessed?; [1]=1 (known onset with ≤60 min uncertainty); [2]= 2 (unknown onset with >60 min uncertainty)"
6. "Wake-up stroke?; [1]=Yes; [2]= No"
7. "In-hospital stroke?; [1]=Yes; [2]= No"
8. "Was patient transferred from another hospital?; [1]= Yes; [2]= No"
9. "IVT administrated at other hospital?; [1]=Yes; [2]=No"
10. "Pre-stroke mRS; number"
11. "Independent prior to stroke; [1]=Yes (=pre-mRS 0-2); [2]=No (pre-mRS >2)"
12. "RFAtrial fibrillation; [1]=Yes; [2]=No"
13. "RF Diabetes; [1]=Yes; [2]=No"
14. "RF Hypertension; [1]=Yes; [2]=No"
15. "RF Hypercholesterolemia; [1]=Yes; [2]=No"
16. "RF current smoking (or stopped <2y); [1]=Yes; [2]=No"
17. "RF coronary artery disease; [1]=Yes; [2]=No"
18. "RF prior ischemic stroke; [1]=Yes; [2]=No"
19. "If prior ischemic stroke yes: prior treatment with; [1]=IVT; [2]=Bridging; [3]=EVT; [4]=No"
20. "Pre-Stroke statins; [1]=Yes; [2]=No"
21. "Pre-Stroke antihypertensive agents; [1]=Yes; [2]=No"
22. "Pre-Stroke antiplatelets; [1]=Yes; [2]=No"
23. "If Pre-Stroke antiplatelets yes: Aspirin; [1]=Yes; [2]=No"
24. "If Pre-Stroke antiplatelets yes: Clopidogrel; [1]=Yes; [2]=No"
25. "If Pre-Stroke antiplatelets yes: Prasugrel; [1]=Yes; [2]=No"
26. "If Pre-Stroke antiplatelets yes: Ticagrelor; [1]=Yes; [2]=No"
27. "If Pre-Stroke antiplatelets yes: Dipyridamol; [1]=Yes; [2]=No"
28. "Pre-IVT Anticoagulants; [1]= No; [2]= Vit K;- [3]= NOAC; [4]= Other"
29. "If NOAC yes: Rivaroxaban; [1]=Yes; [2]=No"
30. "If Rivaroxaban yes: dose in mg; number"
31. "If NOAC yes: Dabigatran; [1]=Yes; [2]=No"
32. "If Dabigatran yes: dose in mg; number"
33. "If NOAC yes: Apixaban; [1]=Yes; [2]=No"
34. "If Apixaban yes: dose in mg: number"
35. "If NOAC yes: Edoxaban; [1]=Yes; [2]=No"
36. "If Edoxoaban yes: dose in mg number"
37. "Epileptic seizure at stroke onset; [1]=Yes; [2]=No"
38. "NIHSS on admission; number"
39. "RR systolic on admission in mmHg; number"
40. "RR diastolic on admission in mmHg; number"
41. "body-weight in kg; number"

42. "height in cm; number"
43. "Creatinine on admission in umol/l; number"
44. "INR prior to IVT; number"
45. "Platelets on admission x10e 9/l; number "
46. "Leucocytes on admission x10e 9/l; number "
47. "Heamoglobin on admission; number "
48. "Glucose on admission; number"
49. "CRP on admission; number"
50. "modified TOAST; [1]= LAA; [2]= CE (incl PFO); [3]= SAO; [4]= Other; [5]= more than one; [6]= undetermined; [7]= stroke mimic"
51. "If Other in modIfied TOAST: Rare specIfic stroke causes to be studied in more detail: artery dissection; [1]=Yes; [2]=No "
52. "If Other in modIfied TOAST: Rare specIfic stroke causes to be studied in more detail: endocarditis; [1]=Yes; [2]=No "
53. "If Other in modIfied TOAST: Rare specIfic stroke causes to be studied in more detail: vasculitis; [1]=Yes; [2]=No"
54. "If Other in modIfied TOAST: Rare specIfic stroke causes to be studied in more detail: coaguopathies; [1]=Yes; [2]=No"
55. "Type of intervention; [0]= none; [1]= IVT; [2]= Bridging; [3]= EVT"
56. "Dosage of rtPA IVT; [1]= Complete; [2]= partial (<50% of dose)"
57. "IVT with Tenecteplase; [1]=Yes; [2]=No"
58. "If tenecteplase: dose in mg/kg; number (0.xx)"
59. "Angioedema related to IVT; [1]=Yes; [2]=No"
60. "Exclusion criteria IVT: time window; [1]=Yes; [2]=No "
61. "Exclusion criteria IVT: major prestroke handicap; [1]=Yes; [2]=No"
62. "Exclusion criteria IVT: imaging contraindication; [1]=Yes; [2]=No"
63. "Exclusion criteria IVT: bleeding risk; [1]=Yes; [2]=No"
64. "Exclusion criteria IVT: missed/uncertain; [1]=Yes; [2]=No"
65. "Exclusion criteria IVT: other; [1]=Yes; [2]=No"
66. "Intraarterial thrombolytic drug; [1]=Yes; [2]=No"
67. "Intra-arterial rtPA dose in mg; number"
68. "General anaesthesia during EVT; [1]=Yes; [2]=No"
69. "Mechanical treatment; [1]=Yes; [2]=No"
70. "If Mechanical treatment yes: stent retriever; [1]=Yes; [2]=No"
71. "If Mechanical treatment yes: aspiration; [1]=Yes; [2]=No"
72. "If Mechanical treatment yes: distal retriever; [1]=Yes; [2]=No"
73. "If Mechanical treatment yes: Intracranial balloon angioplasty; [1]=Yes; [2]=No"
74. "If Mechanical treatment yes: Permanent intracranial stent; [1]=Yes; [2]=No"
75. "If Mechanical treatment yes: Permanent extracranial stent; [1]=Yes; [2]=No"
76. "If Mechanical treatment yes: extracranial thrombectomy; [1]=Yes; [2]=No"
77. "If Mechanical treatment yes: other; [1]=Yes; [2]=No"
78. "Number of device pass or retrieval attempt; number"
79. "Tandem stenosis/occlusion; [1]=Yes; [2]=No"
80. "EVT stopped early; [1]=Yes; [2]=No"
81. "EVT stopped early, reason; [1]= Initiated, but access-to-clot-problems; [2]= Tried, but artery already recanalized; [3]= Other"
82. "EVT complications; [1]=Yes; [2]=No"
83. "If EVT complications yes: vessel perforation; [1]=Yes; [2]=No"
84. "If EVT complications yes: vasospasm; [1]=Yes; [2]=No"
85. "If EVT complications yes: dissection; [1]=Yes; [2]=No"
86. "If EVT complications yes: SAH/ICH; [1]=Yes; [2]=No"
87. "If EVT complications yes: device detachment/misplacement; [1]=Yes; [2]=No"
88. "If EVT complications yes: embolization to new territory; [1]=Yes; [2]=No"
89. "If EVT complications yes: access-site complications; [1]=Yes; [2]=No"
90. "If EVT complications yes: early reocclusion; [1]=Yes; [2]=No"
91. "If EVT complications yes: other; [1]=Yes; [2]=No"

92. "Type of baseline image; [1]= CT; [2]= MR"
93. "Territory of infarction ICA; [1]=Yes; [2]=No "
94. "Territory of infarction MCA; [1]=Yes; [2]=No "
95. "Territory of infarction ACA; [1]=Yes; [2]=No"
96. "Territory of infarction PCA; [1]=Yes; [2]=No"
97. "Territory of infarction Cerebellum; [1]=Yes; [2]=No"
98. "Territory of infarction Brainstem; [1]=Yes; [2]=No"
99. "Side of infarction left anterior circulation; [1]=Yes; [2]=No"
100. "Side of infarction right anterior circulation; [1]=Yes; [2]=No"
101. "Side of infarction posterior circulation; [1]=Yes; [2]=No"
102. "anterior circulation ASPECTS on baseline image; number"
103. "posterior circulation ASPECTS on baseline image; number"
104. "early ischemic changes in suspected area; [1]=Yes; [2]=No"
105. "Occluded vessel with hyperdense artery sign; [1]=Yes; [2]=No"
106. "Type of baseline angiography; [1]= None; [2]= CTA; [3]= MRA"
107. "Site of main intracranial occlusion on baseline angiography: none; [1]=Yes; [2]=No"
108. "Site of main intracranial occlusion on baseline angiography: ICA-I; [1]=Yes; [2]=No"
109. "Site of main intracranial occlusion on baseline angiography: ICA-L/T; [1]=Yes; [2]=No"
110. "Site of main intracranial occlusion on baseline angiography: prox M1; [1]=Yes; [2]=No"
111. "Site of main intracranial occlusion on baseline angiography: distal M1; [1]=Yes; [2]=No"
112. "Site of main intracranial occlusion on baseline angiography: M2; [1]=Yes; [2]=No"
113. "Site of main intracranial occlusion on baseline angiography: ACA; [1]=Yes; [2]=No"
114. "Site of main intracranial occlusion on baseline angiography: PCA; [1]=Yes; [2]=No"
115. "Site of main intracranial occlusion on baseline angiography: BA; [1]=Yes; [2]=No"
116. "Site of main intracranial occlusion on baseline angiography: VA; [1]=Yes; [2]=No"
117. "Site of main intracranial occlusion on baseline angiography: other; [1]=Yes; [2]=No"
118. "In case of ICA/MCA M1/prox M2 occlusions: collaterals on baseline angiography (TAN Score 0-3); number"
119. "If other than TAN coolateral score used: Baseline collaterals; [1]= poor; [2]= intermediate; [3]= good"
120. "Relevant stenosis (>50% NASCET) of extracranial ICA on baseline angiography; [1]=Yes; [2]=No"
121. "Relevant stenosis (>50%) of extracranial VA on baseline angiography; [1]=Yes; [2]=No"
122. "Type of baseline perfusion modality; [1]= None; [2]= CTP; [3]= MRP"
123. "Mismatch ratio visually no infarct core; [1]=Yes; [2]=No"
124. "If no infarct core: no Mismatch ratio visually (f.e. 1.2 = perfusion lesion is 20% larger than infarct core); number"
125. "Mismatch ratio visually >1.8; [1]=Yes; [2]=No"
126. "In case of ICA/MCA M1/prox M2 occlusions: collaterals on DSA (ASITN/SIR grading); number"
127. "Complete recanalization on DSA: [1]=Yes; [2]=No"
128. "Type of first follow-up native image; [1]= None; [2]= NCCT; [3]= MRI"
129. "Type of first follow-up vessel imaging; [1]= None; [2]= CTA; [3]= MRA-TOF; [4]= MRA-CE; [5]= Ultrasound"
130. "Complete recanalization on follow-up vessel imaging (CTA/MRA/US); [1]=Yes; [2]=No"
131. "any ICH; [1]=Yes; [2]=No"
132. "fatal ICH; [1]=Yes; [2]=No"
133. symptomatic ICH (ECASS-2 criteria); [1]=Yes; [2]=No"
134. "symptomatic ICH (ECASS-3 criteria); [1]=Yes; [2]=No"
135. "Other ICH (SAB, SDH etc); [1]=Yes; [2]=No"
136. "NIHSS after 24h; number"
137. "mRS after 3 months; number"
138. "Recurrent ischemic stroke or TIA within 3 months; [1]= No; [2]= ≥1TIA(s); [3]= ≥1stroke(s) "
139. "mRS after 1 year; number"
140. "Discharge destination; [1]= home (no matter if outpatient rehabilitation or not); [2]= rehabilitation clinic (inpatient); [3]= nursing home; [4]= dead; [5]= other acute care hospital"

141. "Length of hospital stay in days; number"
142. "Segments of VAO, (combination possible, separate by comma): [1]= right V0-V3 segment occlusion; [2]= left V0-V3 segment occlusion; [3]= right V4 segment occlusion; [4]= left V4 segment occlusion; [5]= also right PICA occlusion; [6]= also left PICA occlusion"
143. "Stroke clinical specific territory at initial assessment, (combination possible, separate by comma): [0]= asymptomatic; [1]= lateral medullary +/- cerebellar PICA territory*; [2]= medial medullary; [3]= cerebellar PICA territory only**; [4]= brainstem territory other than medullary; [5]= posterior circulation supratentorial territory" *in lateral medullary: any diplopia not clearly attributable to cranial nerve palsy is allowed; contralateral pyramidal signs are also allowed; ** cerebellar PICA territory only: any of the following or combination of (without other signs and symptoms): vertigo, nausea, vomiting, limb ataxia, truncal ataxia
144. "Stroke radiological specific territory on initial imaging, (combination possible, separate by comma): [1]= lateral medullary +/- cerebellar PICA territory; [2]= medial medullary; [3]= cerebellar PICA territory only; [4]= brainstem territory other than medullary; [5]= posterior circulation supratentorial territory; [6]= no radiologically visible lesion"
145. "Recanalization status after EVT (if performed) of V4 segment; [0]= no recanalization; [1]= partial recanalisation 50-99%; [2]= full recanalization; [3]= initially not occluded; [NP]= EVT not performed"
146. "Recanalization status after EVT (if performed) of PICA segment; [0]= no recanalization; [1]= partial recanalisation 50-99%; [2]= full recanalization; [3]= initially not occluded; [NP]= EVT not performed"
147. "Recanalization status after EVT (if performed) of V0-V3 segment; [0]= no recanalization; [1]= partial recanalisation 50-99%; [2]= full recanalization; [3]= initially not occluded; [NP]= EVT not performed"
148. "Recanalization status after follow-up imaging (12-48h) of V4 segment; [0]= no recanalization; [1]= partial recanalisation 50-99%, [2]= full recanalization; [3]= initially not occluded; [NP]= Follow-up imaging not performed"
149. "Recanalization status after follow-up imaging (12-48h) of PICA segment; [0]= no recanalization; [1]= partial recanalisation 50-99%; [2]= full recanalization; [3]= initially not occluded; [NP]=Follow-up imaging not performed"
150. "Recanalization status after follow-up imaging (12-48h) of V0-V3 segment; [0]= no recanalization; [1]= partial recanalisation 50-99%; [2]= full recanalization; [3]= initially not occluded; [NP]=Follow-up imaging not performed"
151. "Patient not treated by IVT and/or EVT because of absence of disabling deficit according to treating physician; [1]= not treated because of non disabling deficit; [2]= not treated because of other reason"
152. DelayLPGH_Admission
153. DelayFound_Admission
154. DelayLPGH_IVT
155. DelayFound_IVT
156. DelayLPGH_Groin
157. DelayFound_Groin
158. DelayAdmiss_BaselineImage
159. DelayGroinRecanal
160. DelayAdmissionFUvessel

Table S5: Missing data of the included patients (N=494)

**Primary and secondary outcome variables**	**Number missing**	**Rate missing**	**Imputed if missing**
3-month mRS	**494/494**	**0%**	No
V4 segment recanalization	**37/381**	**9.7%**	No
ENDi & 24h ΔNIHSS	**30/494**	**6.1%**	No
sICH	**8/494**	**1.6%**	No

**Other variables**	**Number missing**	**Rate missing**	**Imputed if missing**
Age	0/494	0%	Yes
Gender female	0/494	0%	Yes
Year of stroke onset	0/494	0%	No
Onset time known	31/494	6.3%	Yes
Pre-stroke mRS	43/494	8.7%	Yes
Independent prior to stroke	11/494	2.2%	Yes
Atrial fibrillation	0/494	0%	Yes
Diabetes	1/494	0.2%	Yes
Hypertension	0/494	0%	Yes
Dyslipidemia	3/494	0.6%	Yes
Current smoking (or stopped <2y)	5/494	1.0%	Yes
Coronary artery disease	9/494	1.8%	Yes
Prior ischemic stroke	5/494	1.0%	Yes
BMI (Kg/m2)	232/494	47.0%	Yes
Pre-Stroke antiplatelets	34/494	6.9%	Yes
Pre-stroke Anticoagulants	49/494	9.9%	Yes
Pre-stroke statins	59/494	11.9%	Yes
Pre-stroke antihypertensive agents	47/494	9.5%	Yes
Systolic BP on admission (mmHg)	40/494	8.1%	Yes
Diastolic BP on admission (mmHg)	42/494	8.5%	Yes
Creatinine on admission (umol/l)	41/494	8.3%	Yes

Other variables	Number missing	Rate missing	Imputed if missing
Platelets on admission (x10e 9/l)	44/494	8.9%	Yes
Leucocytes on admission (x10e 9/l)	56/494	11.3%	Yes
Hemoglobin on admission	45/494	9.1%	Yes
Blood glucose on admission	43/494	8.7%	Yes
CRP on admission	148/494	30.0%	Yes
NIHSS on admission	5/494	1.0%	Yes
Stroke clinical-radiological localization	4/494	0.8%	Yes
Type of baseline imaging	64/494	13.0%	Yes
Type of baseline vascular imaging	63/494	12.8%	Yes
Posterior circulation ASPECTS on baseline imaging	202/494	40.9%	Yes
Localization of VAO	0/494	0%	Yes
Last seen well to hospital arrival delay (min)	0/494	0%	Yes
Last seen well to needle delay (min)	119/279	42.7%	Yes
Last seen well to groin puncture (min)	50/133	37.6%	Yes
Stroke etiology	5/494	1.0%	Yes
Modality of first follow-up vascular imaging	36/494	7.3%	No
General anesthesia during EVT	32/133	24.1%	No
Stent retriever	35/133	26.3%	No
Aspiration	35/133	26.3%	No
Permanent intracranial stent	37/133	27.8%	No
Permanent extracranial stent	32/133	24.1%	No
Procedural complications	31/133	23.3%	No
Number of device passes	76/133	57.1%	No

Table S6: Assessment for risk of bias

Risk of bias assessment	Possible bias	Overcome measures
**Internal validity**		
Selection bias	A selection bias is possible given the retrospective nature of the study. Treatment allocation was based upon decision of the treating physician at the time of stroke.	Propensity score weighting was used in the statistical analysis.
Information bias	Since a centralized adjudication of imaging was not planned, an information bias on radiological adjudication of specific site of occlusion might exist.	A thorough data curation was performed. Cross-check with other variables was performed on the final dataset and queries were sent in case of inconsistent values. Furthermore, selection of highly specialized centers in the management of acute ischemic stroke should have been able to minimize information bias risk.
Confounding variables	The main variables used for the analysis were variables commonly used in acute ischemic stroke trials and studies. We used the predefined variables collected in the EVA-TRISP consortia projects with additional specific variables that were considered to be specific for the BRAVO project.	For multivariable models, we did not include variables that are known for co-linearity. From a statistical standpoint, multivariate logistic regression models were adopted to account for confounding effect of other variables on the dependent variable.
Loss to follow-up	Given the retrospective nature of the study, it was not possible to overcome such bias. We did not plan to ask centers to recontact patients to obtain an estimation of the 3 month mRS.	People with missing 3m-mRS were excluded from the study (See exclusion flowchart for missing 3m-mRS).
Outcome assessment	Since based on a non-blinded clinical assessment, the primary outcome is possibly subject to an outcome assessment bias.	3m-mRS was performed by certified or trained personnel in each center. Assessment was based on strict definitions able to correctly classify patients according to their functional status.
**External validity**		
Generalizability	Since revascularization treatments are not uniformly available across the world, the results of the study might apply mainly to people from mid-high income countries because of factors such as organization of healthcare system, availability of intravenous thrombolysis or endovascular capable centers	The study is a global multicenter cohort study and tried to include participants from different continents (3).

Table S7: Detailed statistical analysis for IVT vs Cx groups

Table S7a : 3-month favorable shift in ordinal mRS in IVT vs Cx
n=361; Proportional odds assumption met in 10/10 imputed datasets

Variable	OR(95%CI)	P-value
Age	0.97 (0.95-0.99)	0.003
Gender female	0.75 (0.45-1.26)	0.284
Independent prior to stroke	2.46 (1.31-4.61)	0.005
Blood glucose on admission	0.91 (0.85-0.97)	0.004
Clinical-radiological other brainstem lesion	0.92 (0.66-1.27)	0.601
Localization of VAO: Unilateral extracranial	1.82 (1.17-2.84)	0.008
Localization of VAO: Multiple segments occlusion	1.03 (0.60-1.78)	0.902
NIHSS on admission	0.81 (0.76-0.87)	<0.001
TOAST regrouped: Cardioembolic	0.85 (0.40-1.79)	0.674
TOAST regrouped: Other/Undetermined	0.93 (0.59-1.48)	0.774
Last seen well to hospital arrival delay (min): 6-12 hours	0.95 (0.56-1.61)	0.855
Last seen well to hospital arrival delay (min): > 12 hours	0.78 (0.46-1.30)	0.337
Year of stroke onset: 2017-19	1.04 (0.53-2.07)	0.90
Year of stroke onset: 2020-22	0.66 (0.27-1.61)	0.366
IV thrombolysis	1.48 (0.88-2.48)	0.136

Table S7b : MVA binary-analysis for good outcome (0-2) in IVT vs Cx
n=355; Multiple imputation datasets (n=10)

Variable	OR(95%CI)	P-value
Age	0.95 (0.93-0.97)	<0.001
Gender female	0.68 (0.37-1.23)	0.199
Independent prior to stroke	0.65 (0.22-1.94)	0.444
Blood glucose on admission	0.87 (0.81-0.95)	0.001
Clinical-radiological other brainstem lesion	0.75 (0.44-1.27)	0.288
Localization of VAO: Unilateral extracranial	2.31 (1.09-4.91)	0.029
Localization of VAO: Multiple segments occlusion	1.09 (0.54-2.20)	0.808
NIHSS on admission	0.81 (0.74-0.89)	<0.001
TOAST regrouped: Cardioembolic	0.68 (0.30-1.52)	0.343
TOAST regrouped: Other/Undetermined	0.99 (0.57-1.73)	0.983
Last seen well to hospital arrival delay (min): 6-12 hours	0.46 (0.20-1.06)	0.068
Last seen well to hospital arrival delay (min): > 12 hours	0.67 (0.29-1.56)	0.354
Year of stroke onset: 2017-19	1.07 (0.51-2.25)	0.849
Year of stroke onset: 2020-22	0.69 (0.30-1.57)	0.375
IV thrombolysis	1.66 (0.77-3.56)	0.192

Table S7c : MVA binary-analysis for excellent outcome (0-1) in IVT vs Cx
n= 352: Multiple imputation datasets (n=10)

Variable	OR(95%CI)	P-value
Age	0.98 (0.95-1.00)	0.084
Gender female	0.76 (0.42-1.38)	0.371
Independent prior to stroke	0.75 (0.20-2.84)	0.673
Blood glucose on admission	0.95 (0.87-1.04)	0.264
Clinical-radiological other brainstem lesion	1.34 (0.95-1.90)	0.097
Localization of VAO: Unilateral extracranial	2.45 (1.39-4.33)	0.002
Localization of VAO: Multiple segments occlusion	1.11 (0.55-2.23)	0.767
NIHSS on admission	0.82 (0.76-0.89)	<0.001
TOAST regrouped: Cardioembolic	1.08 (0.51-2.27)	0.840
TOAST regrouped: Other/Undetermined	1.23 (0.72-2.11)	0.456
Last seen well to hospital arrival delay (min): 6-12 hours	0.72 (0.43-1.20)	0.210
Last seen well to hospital arrival delay (min): > 12 hours	0.62 (0.31-1.22)	0.163
Year of stroke onset: 2017-19	0.59 (0.25-1.39)	0.230
Year of stroke onset: 2020-22	0.32 (0.11-0.91)	0.032
IV thrombolysis	1.38 (0.85-2.25)	0.191

Table S7d : MVA binary-analysis for partial or complete V4 recanalization in IVT vs Cx

n= 255: Multiple imputation datasets (n=10)

Variable	OR(95%CI)	P-value
Age	1.00 (0.97-1.03)	0.922
Gender female	1.42 (0.57-3.58)	0.454
Localization of VAO: Unilateral extracranial	1.23 (0.28-5.43)	0.786
Localization of VAO: Unilateral intra&extracranial	0.61 (0.35-1.06)	0.081
Localization of VAO: Bilateral intracranial +/- extracranial	1.88 (0.31-11.36)	0.493
Localization of VAO: Other	5.13 (1.04-25.47)	0.045
Modality of first follow-up vascular imaging: CTA	3.00 (1.30-6.92)	0.010
Modality of first follow-up vascular imaging: MRA-TOF	0.99 (0.50-1.95)	0.981
Modality of first follow-up vascular imaging: MRA-CE	2.62 (1.04-6.55)	0.040
Modality of first follow-up vascular imaging: Ultrasound	3.80 (0.87-16.71)	0.077
IV thrombolysis	5.57 (2.10-14.76)	0.001

Table S7e : MVA binary-analysis for early neurological deterioration in IVT vs Cx
n= 341: Multiple imputation datasets (n=10)

Variable	OR(95%CI)	P-value
Age	0.99 (0.95-1.03)	0.548
Gender female	0.55 (0.06-5.27)	0.605
Independent prior to stroke	7e+06 (54.01-8e+11)	0.026
Blood glucose on admission	1.02 (0.86-1.20)	0.835
Clinical-radiological other brainstem lesion	3.08 (1.25-7.58)	0.014
Localization of VAO: Unilateral extracranial	0.00 (0.00-0.00)	<0.001
Localization of VAO: Multiple segments occlusion	1.35 (0.34-5.34)	0.668
NIHSS on admission	1.15 (0.98-1.36)	0.091
TOAST regrouped: Cardioembolic	0.69 (0.15-3.13)	0.632
TOAST regrouped: Other/Undetermined	0.43 (0.07-2.55)	0.351
Last seen well to hospital arrival delay (min): 6-12 hours	1.64 (0.16-16.62)	0.673
Last seen well to hospital arrival delay (min): > 12 hours	1.58 (0.16-15.81)	0.697
Year of stroke onset: 2017-19	1.74 (0.31-9.92)	0.533
Year of stroke onset: 2020-22	1.78 (0.41-7.68)	0.438
IV thrombolysis	1.50 (0.18-12.66)	0.708

Table S7f : MVA binary-analysis for delta NIHSS at 24h in IVT vs Cx
n= 341: Multiple imputation datasets (n=10)

Variable	OR(95%CI)	P-value
Age	0.00 (-0.03-0.03)	0.996
Gender female	0.02 (-0.61-0.64)	0.952
Independent prior to stroke	-0.01 (-1.67-1.65)	0.991
Blood glucose on admission	0.05 (-0.05-0.14)	0.358
Clinical-radiological other brainstem lesion	-0.03 (-0.58-0.51)	0.905
Localization of VAO: Unilateral extracranial	-0.27 (-1.19-0.66)	0.572
Localization of VAO: Multiple segments occlusion	0.19 (-0.66-1.04)	0.667
NIHSS on admission	-0.23 (-0.37--0.09)	0.002
TOAST regrouped: Cardioembolic	-0.12 (-1.27-1.03)	0.842
TOAST regrouped: Other/Undetermined	-0.09 (-0.84-0.66)	0.819
Last seen well to hospital arrival delay (min): 6-12 hours	0.44 (-0.35-1.23)	0.278
Last seen well to hospital arrival delay (min): > 12 hours	0.56 (-0.1-1.22)	0.098
Year of stroke onset: 2017-19	0.20 (-1.32-1.72)	0.80
Year of stroke onset: 2020-22	0.13 (-1.58-1.85)	0.878
IV thrombolysis	-0.54 (-1.07--0.01)	0.045

Table S7g : MVA binary-analysis for symptomatic ICH (ECASS2) in IVT vs Cx
n= 353: Multiple imputation datasets (n=10)

Variable	OR(95%CI)	P-value
Age	1.01 (0.93-1.11)	0.759
Gender female	8.88 (0.30-264.6)	0.207
Independent prior to stroke	7e+08 (NA-NA)	
Blood glucose on admission	1.17 (0.89-1.55)	0.266
Clinical-radiological other brainstem lesion	0.00 (0.00-0.00)	<0.001
Localization of VAO: Unilateral extracranial	0.00 (0.00-0.00)	<0.001
Localization of VAO: Multiple segments occlusion	5.34 (0.63-45.34)	0.125
NIHSS on admission	1.08 (0.86-1.34)	0.517
TOAST regrouped: Cardioembolic	1.81 (0.01-434.9)	0.832
TOAST regrouped: Other/Undetermined	0.64 (0.01-38.67)	0.831
Last seen well to hospital arrival delay (min): 6-12 hours	0.00 (0.00-0.00)	<0.001
Last seen well to hospital arrival delay (min): > 12 hours	0.00 (0.00-0.00)	<0.001
Year of stroke onset: 2017-19	1e+08 (1e+06-9e+09)	<0.001
Year of stroke onset: 2020-22	1e+08 (3e+05-3e+10)	<0.001
IV thrombolysis	4e+07 (5e+06-3e+08)	<0.001

Table S7h : MVA binary-analysis for 3 month mortality in IVT vs Cx
n= 361: Multiple imputation datasets (n=10)

Variable	OR(95%CI)	P-value
Gender female	0.92 (0.31-2.77)	0.889
Independent prior to stroke	0.54 (0.21-1.39)	0.207
Blood glucose on admission	1.19 (1.05-1.36)	0.008
Clinical-radiological other brainstem lesion	1.37 (0.62-3.02)	0.439
Localization of VAO: Unilateral extracranial	0.59 (0.24-1.46)	0.254
Localization of VAO: Multiple segments occlusion	1.33 (0.55-3.22)	0.521
NIHSS on admission	1.25 (1.12-1.40)	<0.001
TOAST regrouped: Cardioembolic	4.58 (1.65-12.72)	0.003
TOAST regrouped: Other/Undetermined	2.58 (0.87-7.66)	0.089
Last seen well to hospital arrival delay (min): 6-12 hours	0.57 (0.14-2.27)	0.426
Last seen well to hospital arrival delay (min): > 12 hours	0.95 (0.36-2.49)	0.911
Year of stroke onset: 2017-19	1.13 (0.36-3.50)	0.837
Year of stroke onset: 2020-22	1.12 (0.37-3.42)	0.844
IV thrombolysis	0.48 (0.19-1.25)	0.135

Table S8: Detailed statistical analysis for EVT±IVT vs MM groups

Table S8a : MVA shift-analysis in EVT±IVT vs MM
n=494; Proportional odds assumption met in 10/10 imputed datasets

Variable	OR(95%CI)	P-value
Age	0.97 (0.95-0.98)	<0.001
Gender female	0.84 (0.55-1.29)	0.425
Independent prior to stroke	2.69 (1.55-4.66)	<0.001
Blood glucose on admission	0.90 (0.86-0.95)	<0.001
Clinical-radiological other brainstem lesion	0.91 (0.66-1.24)	0.536
Localization of VAO: Unilateral extracranial	1.79 (1.24-2.60)	0.002
Localization of VAO: Multiple segments occlusion	0.99 (0.64-1.53)	0.964
NIHSS on admission	0.86 (0.81-0.90)	<0.001
TOAST regrouped: Cardioembolic	0.94 (0.65-1.37)	0.761
TOAST regrouped: Other/Undetermined	0.93 (0.64-1.35)	0.697
IV thrombolysis	1.03 (0.71-1.49)	0.881
Last seen well to hospital arrival delay (min): 6-12 hours	0.75 (0.49-1.16)	0.203
Last seen well to hospital arrival delay (min): > 12 hours	0.70 (0.46-1.06)	0.094
Year of stroke onset: 2017-19	1.04 (0.56-1.96)	0.891
Year of stroke onset: 2020-22	0.58 (0.28-1.20)	0.144
Endovascular treatment	0.59 (0.40-0.85)	0.005

Table S8b : MVA binary-analysis for good outcome (0-2) in EVT±IVT vs MM
n= 478; Multiple imputation datasets (n=10)

Variable	OR(95%CI)	P-value
Age	0.95 (0.94-0.97)	<0.001
Gender female	0.77 (0.45-1.31)	0.333
Independent prior to stroke	0.89 (0.34-2.36)	0.818
Blood glucose on admission	0.86 (0.80-0.93)	<0.001
Clinical-radiological other brainstem lesion	0.84 (0.54-1.33)	0.463
Localization of VAO: Unilateral extracranial	2.20 (1.22-3.95)	0.009
Localization of VAO: Multiple segments occlusion	0.98 (0.58-1.64)	0.924
NIHSS on admission	0.85 (0.80-0.90)	<0.001
TOAST regrouped: Cardioembolic	0.83 (0.49-1.38)	0.463
TOAST regrouped: Other/Undetermined	0.88 (0.59-1.32)	0.542
IV thrombolysis	1.28 (0.67-2.44)	0.457
Last seen well to hospital arrival delay (min): 6-12 hours	0.44 (0.24-0.80)	0.008
Last seen well to hospital arrival delay (min): > 12 hours	0.59 (0.30-1.18)	0.139
Year of stroke onset: 2017-19	0.94 (0.54-1.64)	0.826
Year of stroke onset: 2020-22	0.48 (0.23-1.02)	0.058
Endovascular treatment	0.75 (0.44-1.26)	0.270

Table S8c : MVA binary-analysis for excellent outcome (0-1) in EVT±IVT vs MM
n= 469; Multiple imputation datasets (n=10)

Variable	OR(95%CI)	P-value
Age	0.98 (0.96-1.00)	0.022
Gender female	0.69 (0.43-1.12)	0.133
Independent prior to stroke	0.83 (0.24-2.85)	0.773
Blood glucose on admission	0.94 (0.87-1.01)	0.089
Clinical-radiological other brainstem lesion	1.54 (1.17-2.02)	0.002
Localization of VAO: Unilateral extracranial	2.33 (1.50-3.61)	<0.001
Localization of VAO: Multiple segments occlusion	1.10 (0.61-1.99)	0.753
NIHSS on admission	0.86 (0.81-0.90)	<0.001
TOAST regrouped: Cardioembolic	1.27 (0.70-2.29)	0.432
TOAST regrouped: Other/Undetermined	1.24 (0.78-1.95)	0.363
IV thrombolysis	1.07 (0.68-1.68)	0.771
Last seen well to hospital arrival delay (min): 6-12 hours	0.83 (0.49-1.40)	0.478
Last seen well to hospital arrival delay (min): > 12 hours	0.53 (0.29-0.97)	0.039
Year of stroke onset: 2017-19	0.66 (0.35-1.25)	0.207
Year of stroke onset: 2020-22	0.31 (0.13-0.73)	0.007
Endovascular treatment	0.54 (0.30-0.95)	0.034

Table S8d : MVA binary-analysis for partial or complete V4 recanalization in EVT±IVT vs MM

n=272; Multiple imputation datasets (n=10)

Variable	OR(95%CI)	P-value
Age	1.00 (0.98-1.02)	0.816
Gender female	1.21 (0.61-2.39)	0.588
Localization of VAO: Unilateral extracranial	0.78 (0.13-4.64)	0.787
Localization of VAO: Unilateral intra&extracranial	0.72 (0.45-1.16)	0.178
Localization of VAO: Bilateral intracranial +/- extracranial	1.43 (0.35-5.94)	0.618
Localization of VAO: Other	2.00 (0.30-13.3)	0.475
Modality of first follow-up vascular imaging: CTA	1.68 (0.85-3.30)	0.134
Modality of first follow-up vascular imaging: MRA-TOF	0.59 (0.27-1.29)	0.186
Modality of first follow-up vascular imaging: MRA-CE	1.20 (0.57-2.54)	0.628
Modality of first follow-up vascular imaging: Ultrasound	1.59 (0.59-4.25)	0.358
IV thrombolysis	2.23 (1.18-4.21)	0.014
Endovascular treatment	5.22 (2.35-11.58)	<0.001

Table S8e : MVA binary-analysis for early neurological deterioration in EVT±IVT vs MM

n= 463; Multiple imputation datasets (n=10)

Variable	OR(95%CI)	P-value
Age	1.01 (0.98-1.04)	0.566
Gender female	0.99 (0.38-2.57)	0.981
Independent prior to stroke	1.20 (0.12-12.11)	0.875
Blood glucose on admission	1.00 (0.89-1.12)	0.981
Clinical-radiological other brainstem lesion	1.08 (0.33-3.50)	0.90
Localization of VAO: Unilateral extracranial	0.25 (0.07-0.85)	0.027
Localization of VAO: Multiple segments occlusion	1.29 (0.58-2.86)	0.525
NIHSS on admission	0.98 (0.91-1.06)	0.620
TOAST regrouped: Cardioembolic	0.74 (0.40-1.36)	0.328
TOAST regrouped: Other/Undetermined	0.70 (0.25-1.94)	0.495
IV thrombolysis	2.59 (1.34-5.01)	0.005
Last seen well to hospital arrival delay (min): 6-12 hours	0.39 (0.09-1.70)	0.212
Last seen well to hospital arrival delay (min): > 12 hours	1.42 (0.56-3.62)	0.456
Year of stroke onset: 2017-19	1.51 (0.66-3.46)	0.326
Year of stroke onset: 2020-22	1.42 (0.69-2.91)	0.338
Endovascular treatment	11.04 (2.74-44.42)	0.001

**Table S8f : MVA binary-analysis for delta NIHSS at 24h in EVT±IVT vs MM**<br>n= 463; Multiple imputation datasets (n=10)		
Variable	OR(95%CI)	P-value
Age	0.01 (-0.02-0.04)	0.508
Gender female	-0.09 (-0.76-0.58)	0.789
Independent prior to stroke	0.02 (-1.34-1.39)	0.975
Blood glucose on admission	0.06 (-0.02-0.13)	0.139
Clinical-radiological other brainstem lesion	-0.08 (-0.63-0.47)	0.786
Localization of VAO: Unilateral extracranial	-0.25 (-10-0.49)	0.504
Localization of VAO: Multiple segments occlusion	0.18 (-0.55-0.91)	0.636
NIHSS on admission	-0.21 (-0.37--0.05)	0.011
TOAST regrouped: Cardioembolic	0.07 (-0.82-0.95)	0.882
TOAST regrouped: Other/Undetermined	-0.01 (-0.55-0.53)	0.974
IV thrombolysis	-0.57 (-1.19-0.05)	0.072
Last seen well to hospital arrival delay (min): 6-12 hours	0.19 (-0.66-1.04)	0.664
Last seen well to hospital arrival delay (min): > 12 hours	0.34 (-0.33-1.01)	0.320
Year of stroke onset: 2017-19	0.26 (-0.8-1.31)	0.633
Year of stroke onset: 2020-22	0.28 (-0.8-1.37)	0.609
Endovascular treatment	0.72 (-10-2.44)	0.412

Table S8g : MVA binary-analysis for symptomatic ICH (ECASS2) in EVT±IVT vs MM
n= 485; Multiple imputation datasets (n=10)

Variable	OR(95%CI)	P-value
Age	1.05 (0.98-1.12)	0.161
Gender female	1.61 (0.19-13.71)	0.663
Independent prior to stroke	2e+07 (2e+06-1e+08)	<0.001
Blood glucose on admission	1.05 (0.86-1.29)	0.635
Clinical-radiological other brainstem lesion	2.02 (0.36-11.51)	0.427
Localization of VAO: Unilateral extracranial	0.00 (0.00-0.00)	<0.001
Localization of VAO: Multiple segments occlusion	2.40 (0.57-10.18)	0.234
NIHSS on admission	0.80 (0.64-1.00)	0.051
TOAST regrouped: Cardioembolic	2.42 (0.53-10.94)	0.252
TOAST regrouped: Other/Undetermined	1.41 (0.34-5.89)	0.634
IV thrombolysis	4.42 (0.82-23.91)	0.085
Last seen well to hospital arrival delay (min): 6-12 hours	2.14 (1.06-4.31)	0.033
Last seen well to hospital arrival delay (min): > 12 hours	0.00 (0.00-0.00)	<0.001
Year of stroke onset: 2017-19	2.80 (0.61-12.82)	0.185
Year of stroke onset: 2020-22	3.15 (0.82-12.12)	0.096
Endovascular treatment	7.12 (1.77-28.67)	0.006

Table S8h : MVA binary-analysis for 3 month mortality in EVT±IVT vs MM
n= 494; Multiple imputation datasets (n=10)

Variable	OR(95%CI)	P-value
Age	1.05 (1.02-1.08)	0.001
Gender female	0.71 (0.33-1.52)	0.375
Independent prior to stroke	0.46 (0.19-1.08)	0.076
Blood glucose on admission	1.15 (1.04-1.28)	0.005
Clinical-radiological other brainstem lesion	1.73 (0.89-3.38)	0.109
Localization of VAO: Unilateral extracranial	0.53 (0.27-1.07)	0.078
Localization of VAO: Multiple segments occlusion	1.18 (0.45-3.10)	0.740
NIHSS on admission	1.16 (1.07-1.25)	<0.001
TOAST regrouped: Cardioembolic	2.61 (1.09-6.21)	0.031
TOAST regrouped: Other/Undetermined	2.32 (1.04-5.19)	0.041
IV thrombolysis	1.23 (0.69-2.21)	0.484
Last seen well to hospital arrival delay (min): 6-12 hours	1.76 (0.77-4.02)	0.183
Last seen well to hospital arrival delay (min): > 12 hours	1.37 (0.58-3.27)	0.474
Year of stroke onset: 2017-19	1.31 (0.61-2.80)	0.488
Year of stroke onset: 2020-22	1.91 (0.94-3.91)	0.075
Endovascular treatment	1.60 (0.88-2.93)	0.126

Table S9: Detailed characteristics of mechanical treatment procedure

Center	Aspiration alone	Stent-retriever alone	Combined Aspir & Stent-retr	Ballon angioplasty	Permanent stent
Asan Medical Center<br>*Seoul, South Korea*	1/5	2/5	0/5	4/5<br>(2 w/o asp./stentr.)	3/5<br>(1 w/o asp./stentr.)
St. John's Hospital<br>*Vienna, Austria*	1/2	0/2	1/2	0/2	0/2
University Hospital Basel<br>*Basel, Switzerland*	1/3	0/3	2/3	0/3	1/3
University Clinical Centre of Serbia<br>*Belgrade, Serbia*	1/12	7/12	4/12	0/12	0/12
Charité-Universitätsmedizin Berlin<br>*Berlin, Germany*	3/6	3/6	0/6	2/6	0/6
University Hospital Bern<br>*Bern, Switzerland*	1/2	0/2	1/2	0/2	0/2
Maggiore Hospital<br>*Bologna, Italy*	2/7	0/7	5/7	2/7	2/7
University Hospital of Brescia<br>*Brescia, Italy*	4/13	6/13	3/13	0/13	0/13
Cooper University Hospital<br>*Camden, United States*	0/1	0/1	0/1	0/1	0/1
Duke University School of Medicine<br>*Durham, United States*	0/1	1/1	1/1	0/1	0/1
Hadassah-Hebrew Univ. Medical Center<br>*Jerusalem, Israel*	0/1	1/1	0/1	1/1	1/1
Sahlgrenska University Hospital<br>*Göteborg, Sweden*	6/7	0/7	1/7	1/7	2/7
University Medical Center Hamburg-Eppendorf<br>*Hamburg, Germany*	2/7	1/7	4/7	2/7	3/7
McGovern Medical School<br>*Houston, Texas*	1/7	0/7	5/7	2/7<br>(1 w/o asp./stentr.)	2/7<br>(1 w/o asp./stentr.)
Lausanne University Hospital<br>*Lausanne, Switzerland*	5/11	6/11	0/11	3/11	2/11
Centre Hospitalier Universitaire Lille<br>*Lille, France*	0/1	0/1	1/1	0/1	1/1
Hospital de Egas Moniz<br>*Lisbon, Portugal*	1/1	0/1	0/1	0/1	0/1
Michigan State University<br>*Grand Rapids, United States*	0/2	1/2	1/2	0/1	0/1
Azienda Ospedaliero-Univ. Modena<br>*Modena, Italy*	N/A				
Ludwig-Maximilians-Universitat<br>*Munchen, Germany*	0/5	0/5	4/5	3/5<br>(1 w/o asp./stentr.)	3/5<br>(1 w/o asp./stentr.)
IRCCS di Reggio Emilia<br>*Reggio Emilia, Italy*	1/1	0/1	0/1	0/1	0/1
**TOTAL**	**30/95 (32%)**	**28/95 (30%)**	**33/95 (35%)**	**20/95 (21%)**	**20/95 (21%)**

Detailed characteristics of mechanical treatment procedure

Table S10: Detailed procedural complications of mechanical treatment

**Complication**	**Number of patients experiencing a complication**
Vessel perforation	1
Vasospasm	2
Arterial dissection	3
Subarachnoid/Intracranial hemorrhage	0
Device failure (detachment/misplacement)	0
Embolization to previously non-ischemic territory	4
Acces-site complications	0
Early re-occlusions	3
Multiple	4, of which :<br>- 1 vessel perforation, embolization in new territory and early re-occlusion<br>- 1 vessel perforation and embolization in new territory<br>- 1 embolization in new territory and access-site complications<br>- 1 multiple not detailed
**TOTAL**	**17/133 (12.8%)**

Detailed characteristics of procedural complications of mechanical treatment

Figure S1: Exclusion flowchart

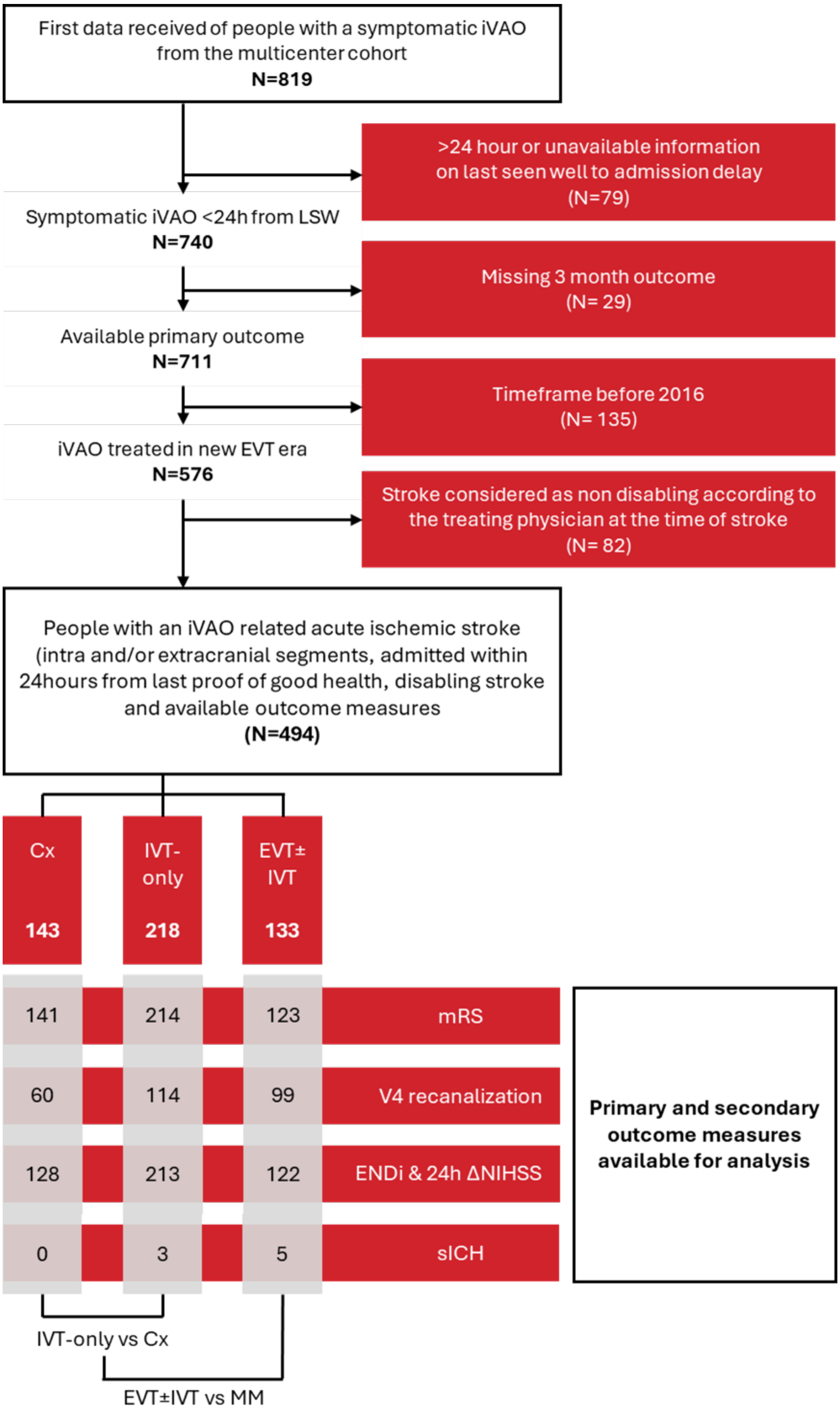

Exclusion flowchart from initial data of 740 people respecting inclusion criterias provided by the centers. Final analyses were performed on data from 494 people after exclusion as described in the chart.

Figure S2: Severity-Outcome analysis for EVT vs MM good outcome

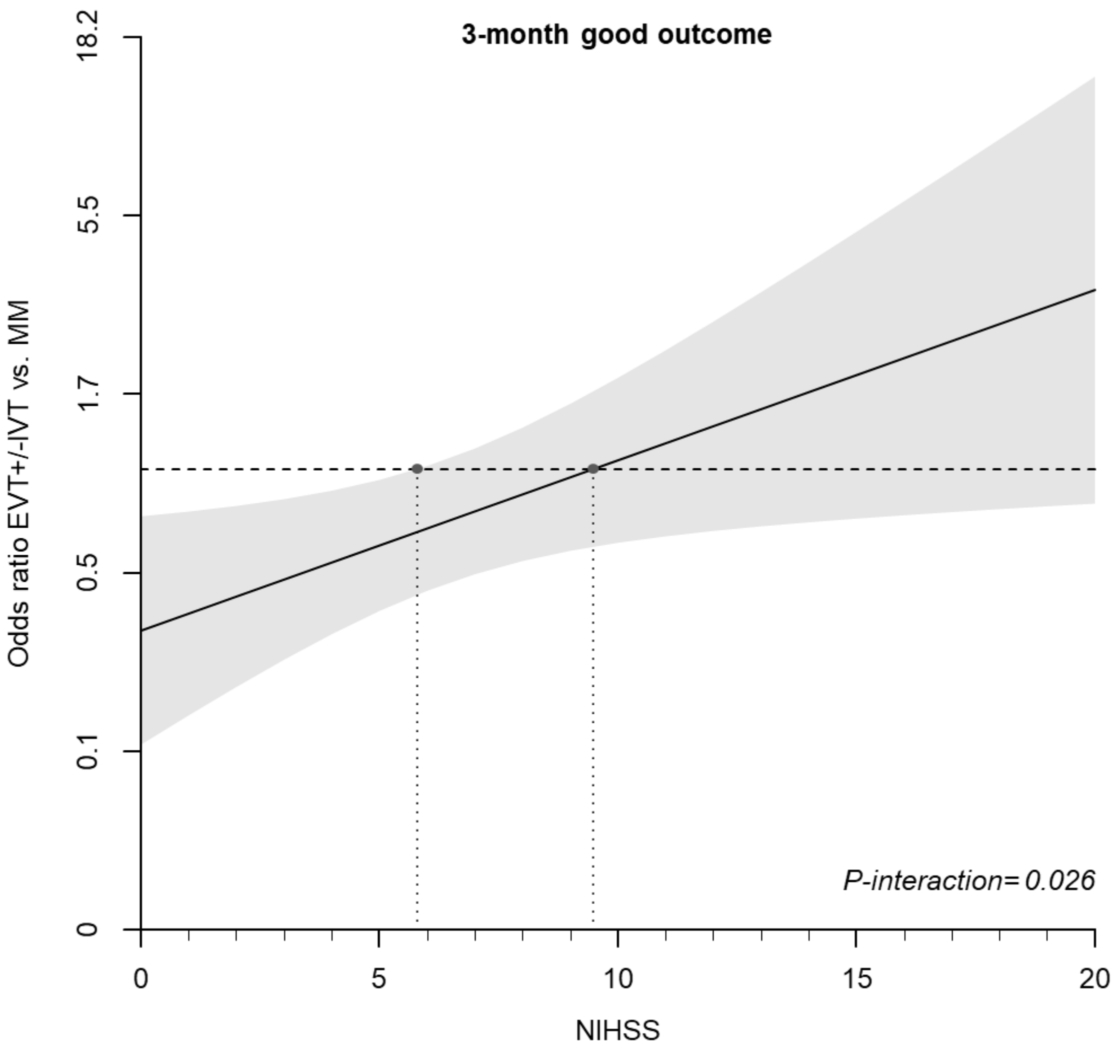

Plot of OR for 3month good outcome (0-2) mRS towards admission NIHSS according to treatment in the EVT+/-IVT vs MM analysis.

Figure S3: Severity-Outcome analysis for EVT vs MM excellent outcome

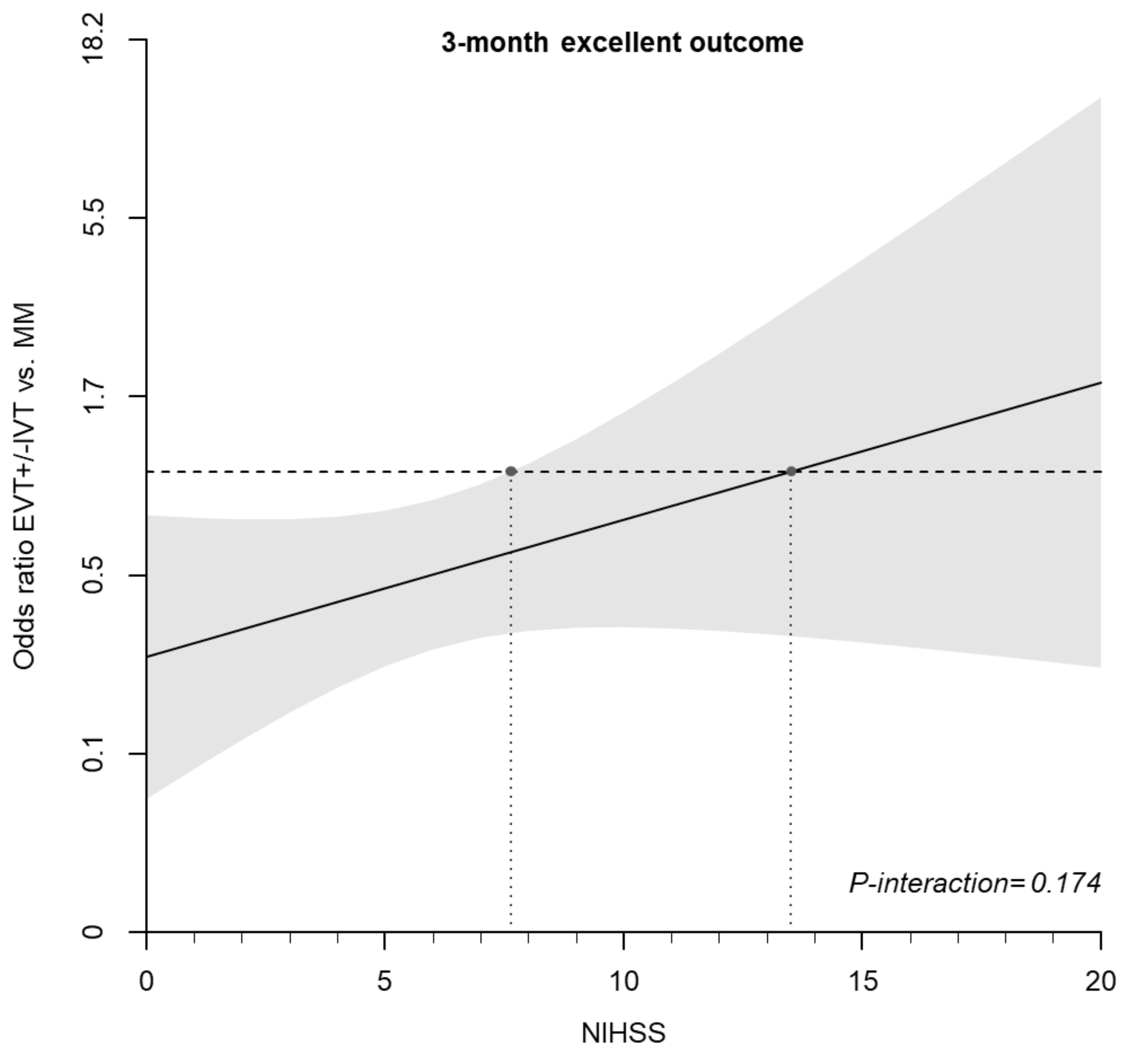

Plot of OR for 3month excellent outcome (0-1) mRS towards admission NIHSS according to treatment in the EVT+/-IVT vs MM analysis.

Figure S4: Severity-Outcome analysis for EVT vs MM mortality

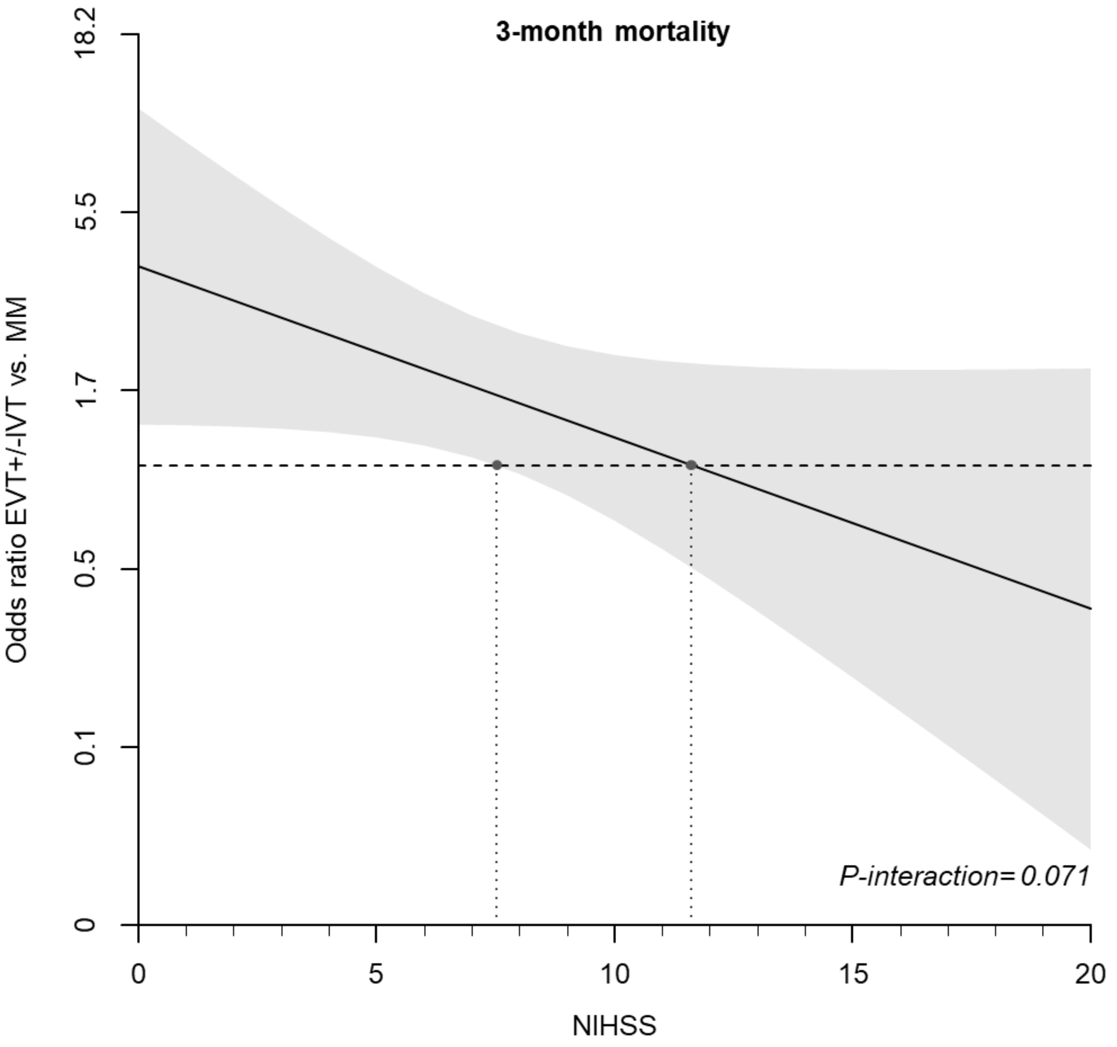

Plot of OR for 3month mortality towards admission NIHSS according to treatment in the EVT+/-IVT vs MM analysis.

Figure S5: Spearman correlation between ENDi severity described in terms of NIHSS point degradation and 3-month disability within the EVT subgroup

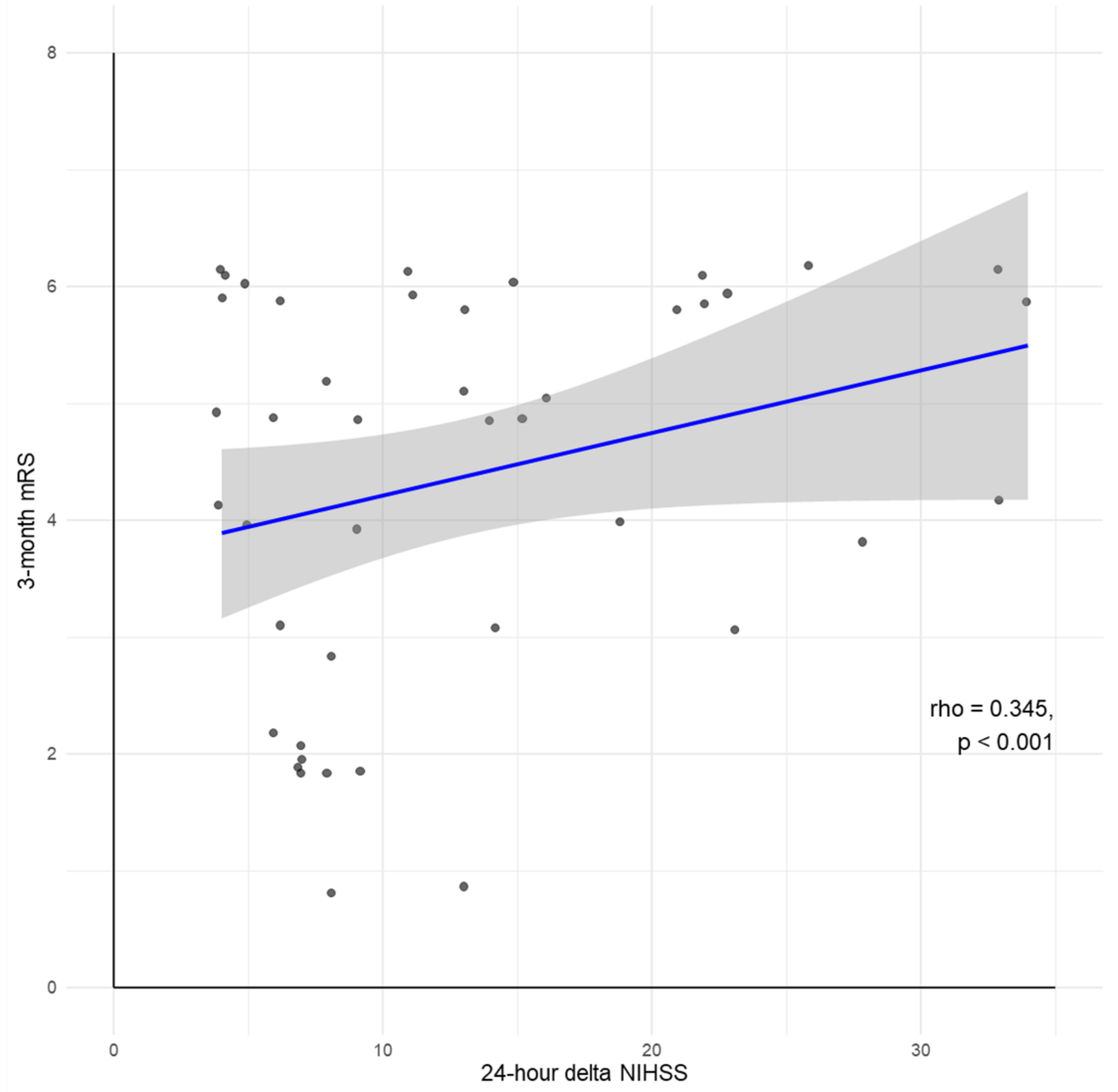

Scatterplot of the correlation between early neurological deterioration (ENDi) severity and 3-month functional outcome in the EVT subgroup.

Figure S6: Standardized mean difference (SMD) for multiple imputed datasets (Unweighted vs. Weighted)

Figure S6A: Standardized mean difference (SMD) for multiple imputed datasets (Unweighted vs. Weighted) in the disability models for the IVT cohort

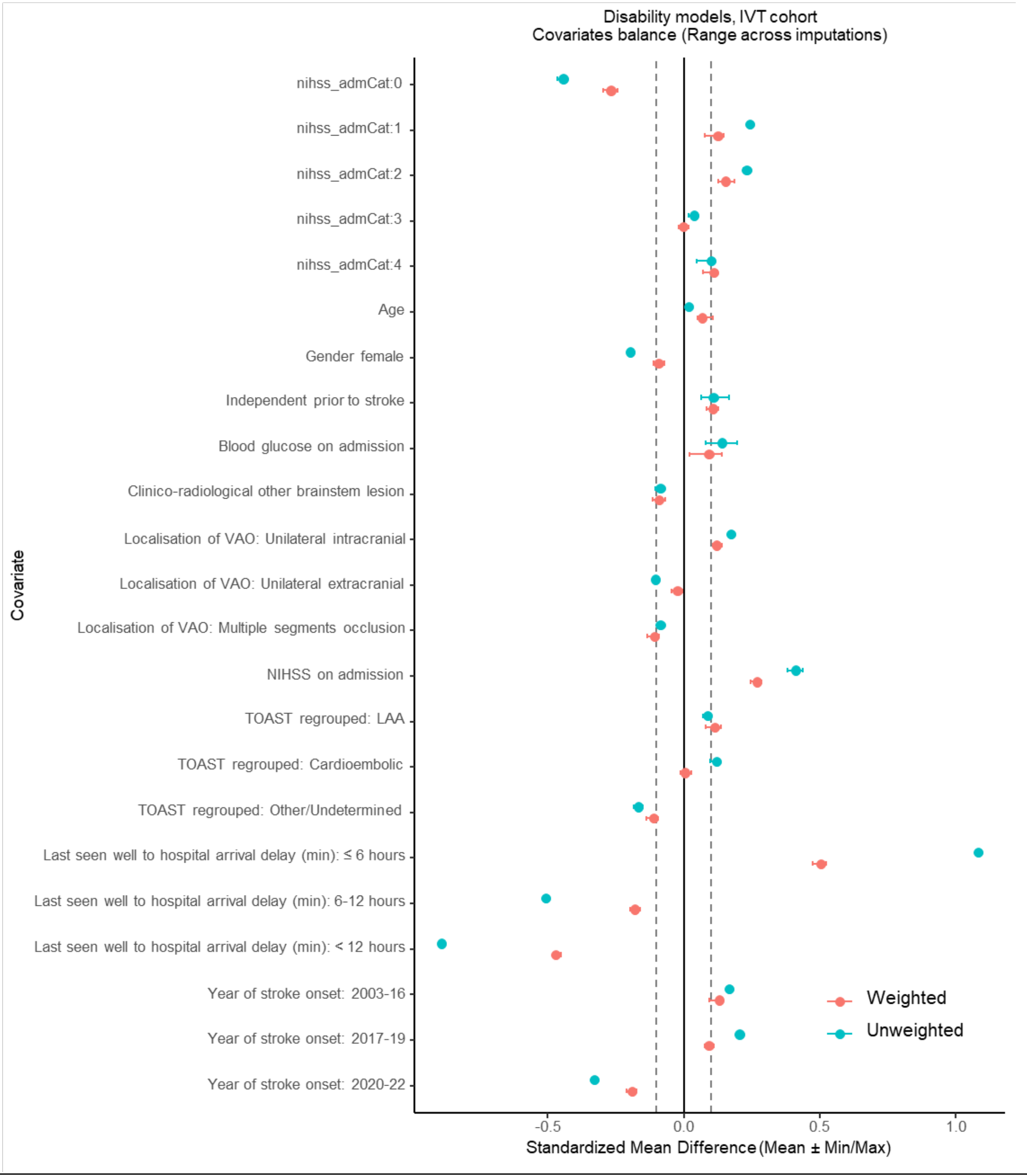

Figure S6B: Standardized mean difference (SMD) for multiple imputed datasets (Unweighted vs. Weighted) in the disability models for the EVT cohort

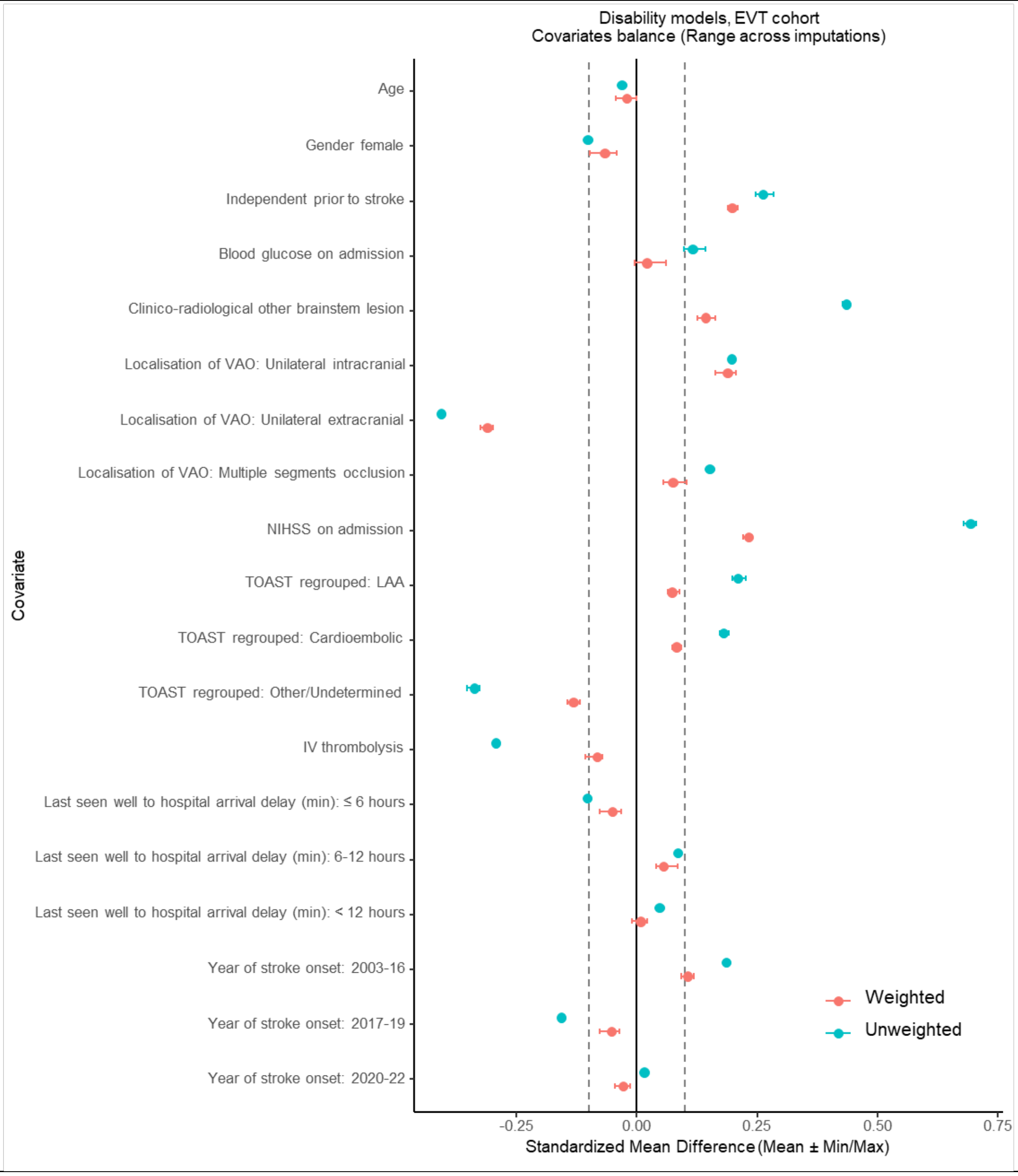

Figure S6C: Standardized mean difference (SMD) for multiple imputed datasets (Unweighted vs. Weighted) in the recanalization models for the IVT cohort

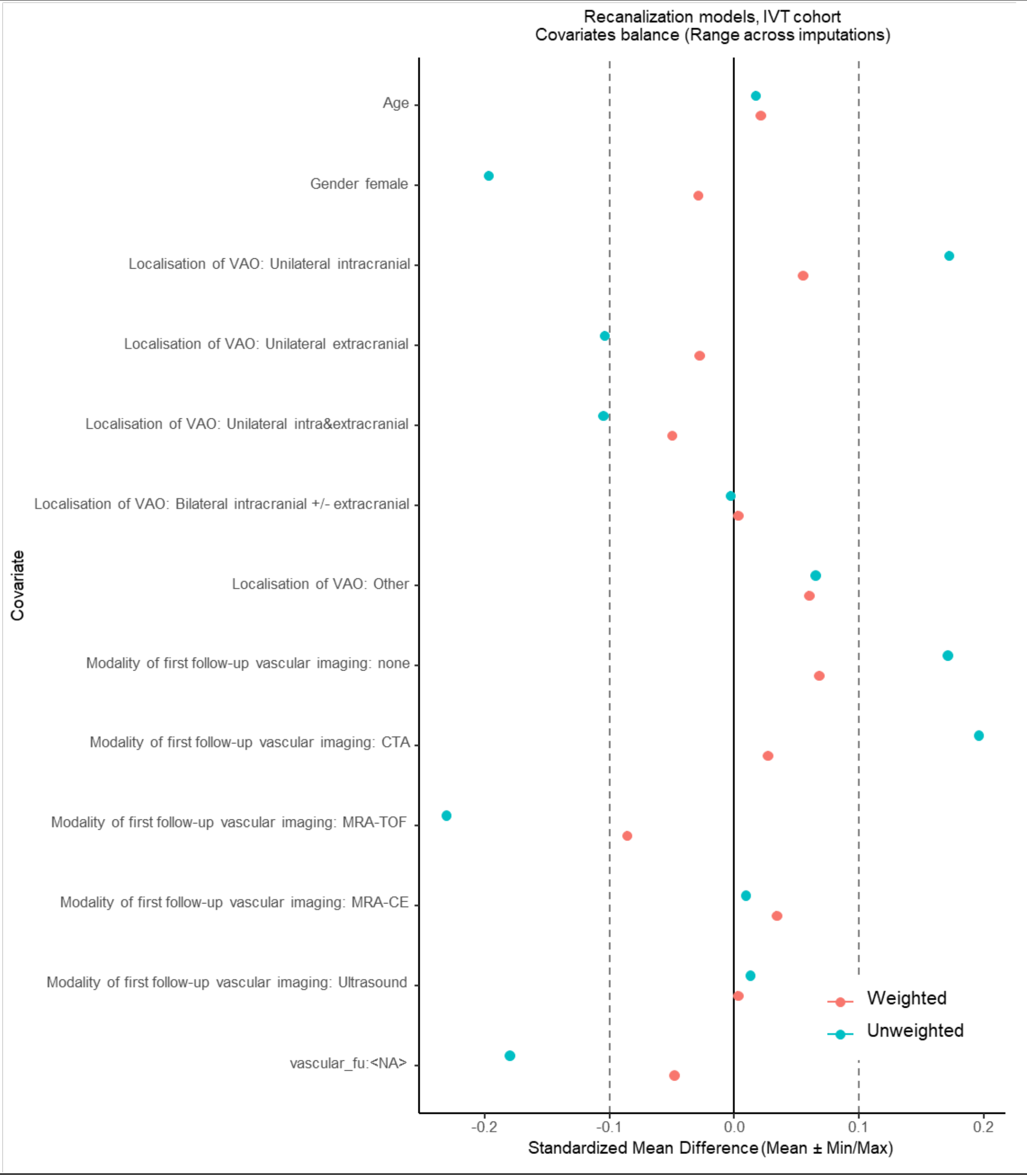

Figure S6D: Standardized mean difference (SMD) for multiple imputed datasets (Unweighted vs. Weighted) in the recanalization models for the EVT cohort

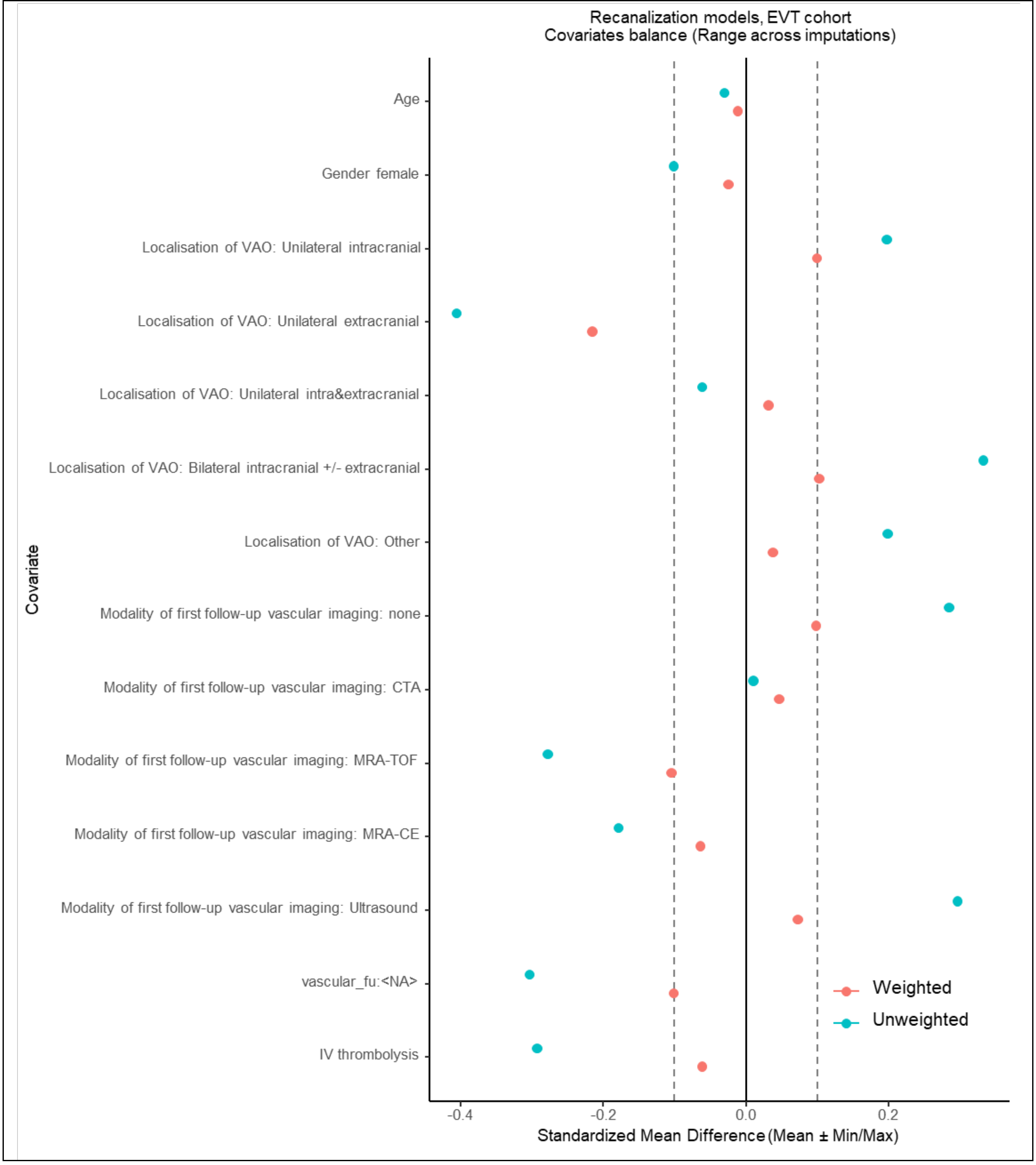

Figure S7: Propensity score distribution in the models

Figure S7A: Propensity score distribution in the disability model for the IVT cohort

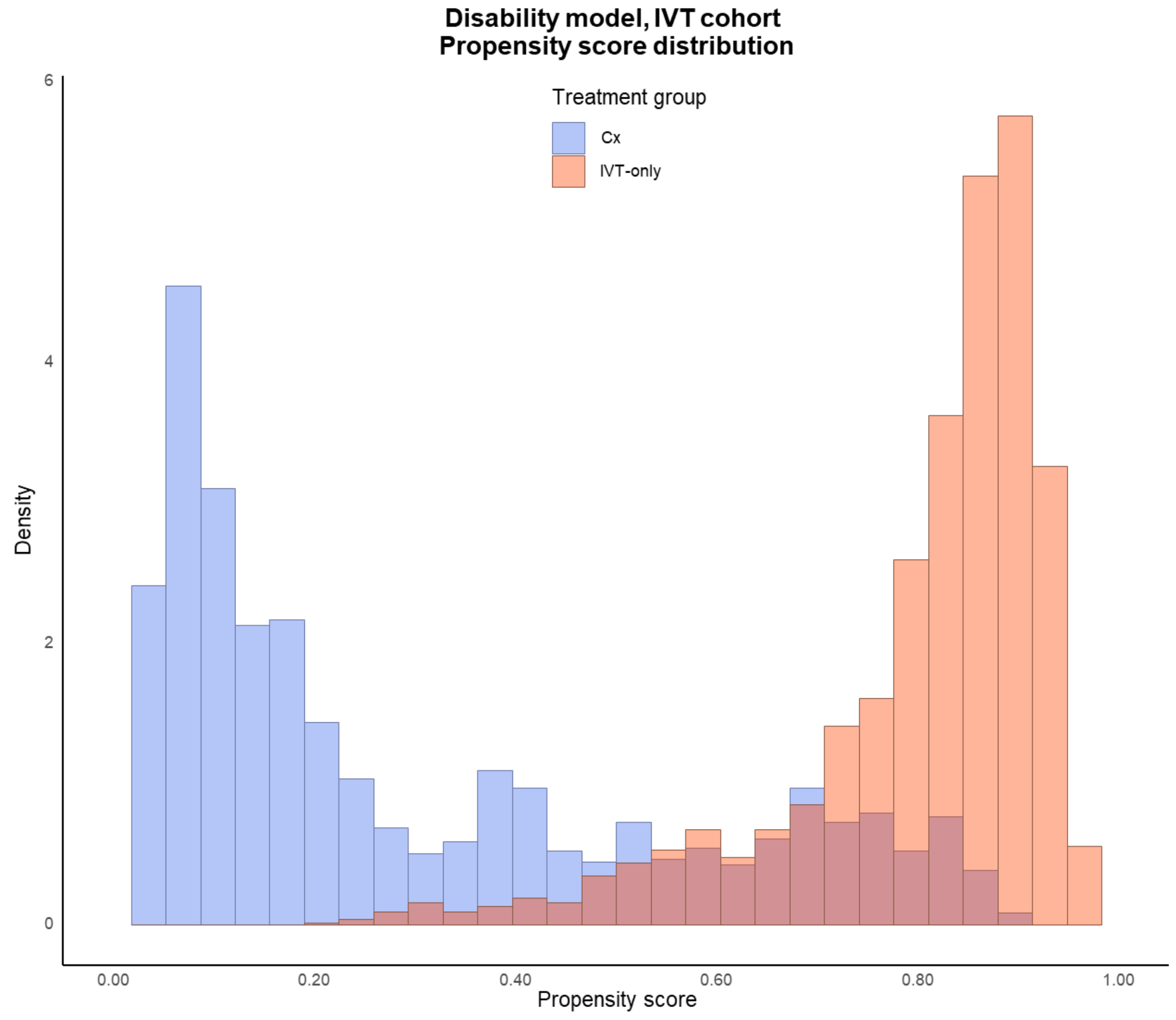

Figure S7B: Propensity score distribution in the recanalization model for the IVT cohort

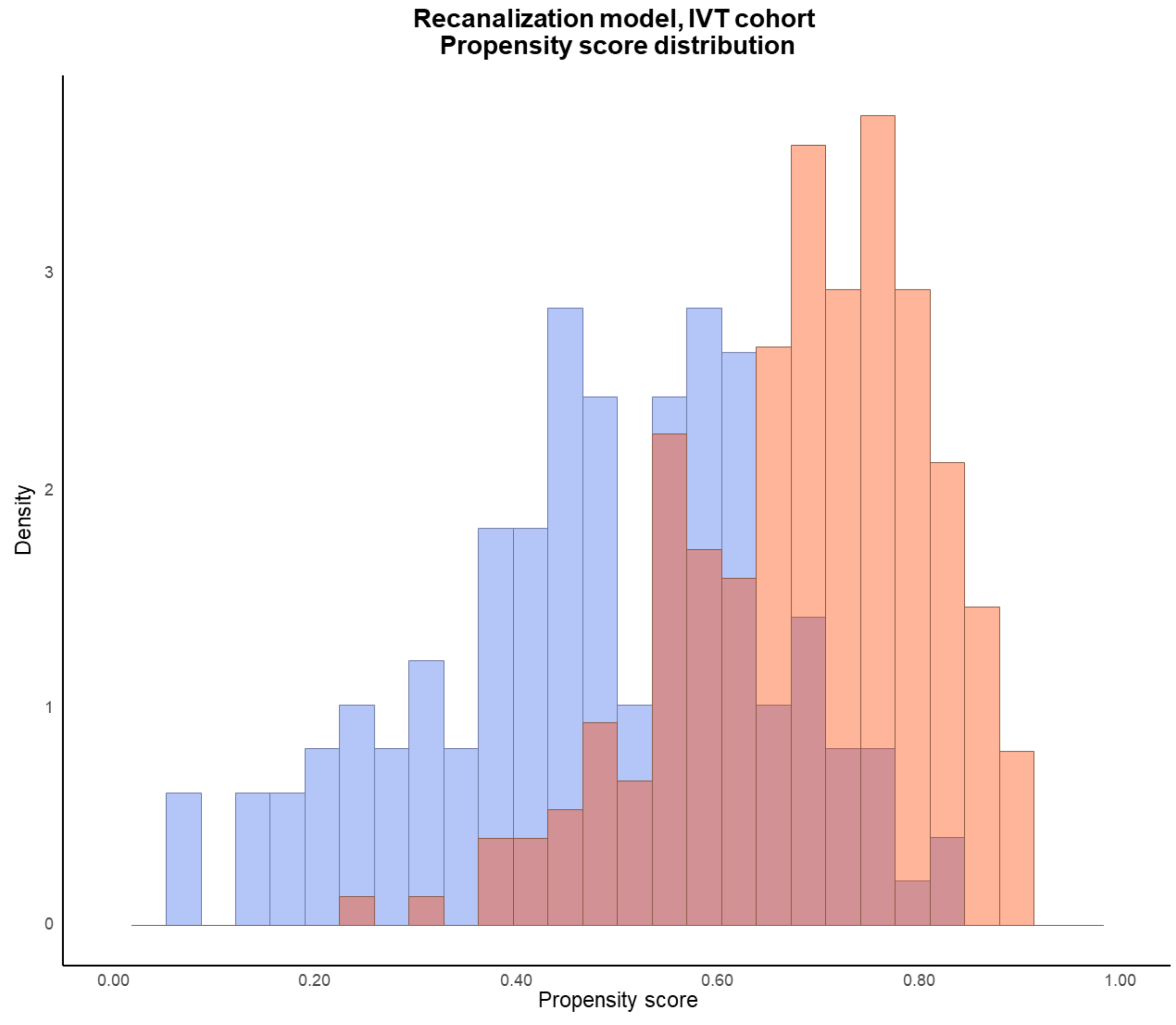

Figure S7C: Propensity score distribution in the disability model for the EVT cohort

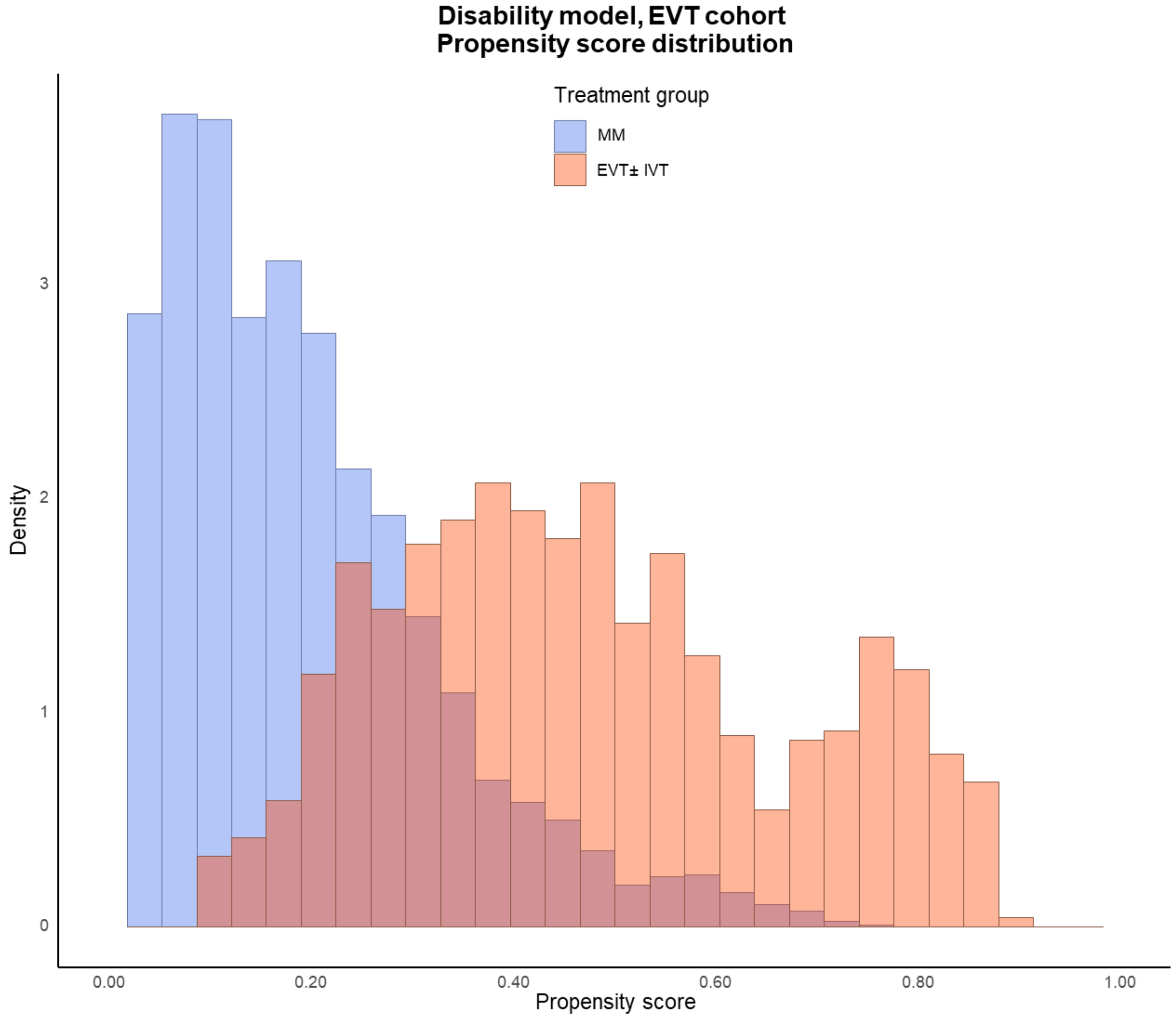

Figure S7D: Propensity score distribution in the recanalization model for the EVT cohort

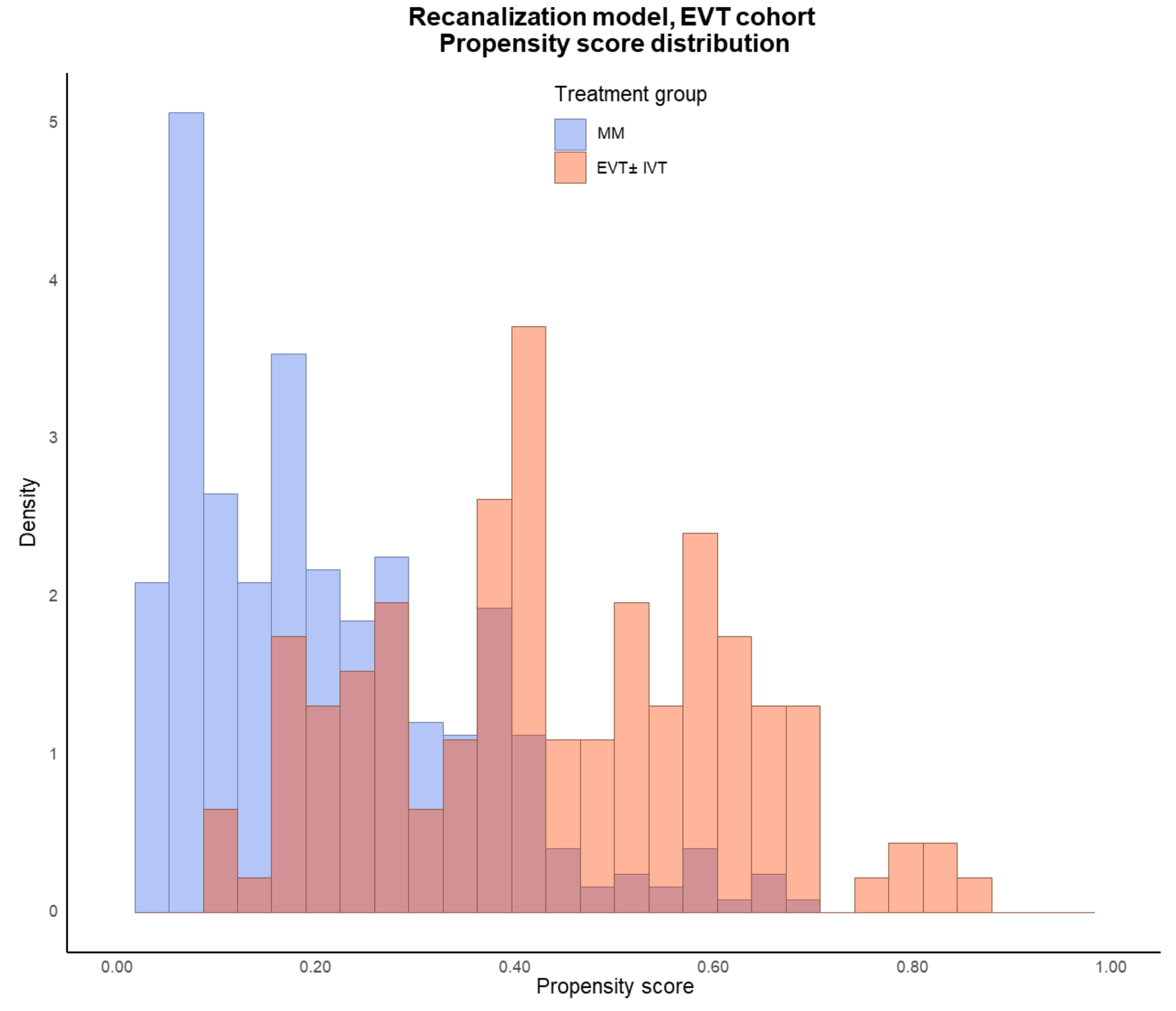

Supplement: Supplementary file 1 [file str-57-1149-s001.pdf]
